# *In vitro* antiplasmodial activity and toxicological profile of extracts, fractions and chemical constituents of leaves and stem bark from *Dacryodes edulis* (Burseraceae)

**DOI:** 10.1186/s12906-023-03957-2

**Published:** 2023-06-27

**Authors:** Kevine Johane Jumeta Dongmo, Mariscal Brice Tchatat Tali, Yannick Stéphane Fotsing Fongang, Pierre Leonel K. Tafokeu Taguimjeu, Donald Ulrich Kenou Kagho, Gabin Thierry Bitchagno, Bruno Ndjakou Lenta, Fabrice Fekam Boyom, Norbert Sewald, Silvère Augustin Ngouela

**Affiliations:** 1 Department of Organic Chemistry, Faculty of Science, University of Yaoundé I, P.O. Box 812, Yaoundé, Cameroon; 2 Antimicrobial and Biocontrol Agents Unit, Faculty of Science, University of Yaoundé I, P.O. Box 812, Yaoundé, Cameroon; 3 Department of Chemistry, Higher Teachers’ Training College, The University of Maroua, P.O. Box 55, Maroua, Cameroon; 4 Department of Chemistry, University of Dschang, P.O. Box 67, Dschang, Cameroon; 5 Department of Chemistry, Higher Teacher Training College, University of Yaoundé I, P.O. Box 47, Yaoundé, Cameroon; 6 Department of Chemistry, Bielefeld University, P.O. Box 100131, 33501 Bielefeld, Germany

**Keywords:** *Dacryodes edulis*, Burseraceae, Antiplasmodial, Acute toxicity, Cytotoxicity, 3,3′,4-tri-*O*-methylellagic acid

## Abstract

**Background:**

*Dacryodes edulis* is a plant that belongs to the Burseraceae family. It is widely used traditionally alone or in association with other plants in Cameroonian folk medicine to cure wounds, fever, headaches, and malaria. The aim of this work was to investigate the leaves and stem bark of *D. edulis* with an emphasis on the antiplasmodial and cytotoxic effects of extracts, fractions, and isolated compounds.

**Methods:**

Extracts, fractions, and some isolated compounds were subjected to antiplasmodial activity screening in vitro against chloroquine-sensitive 3D7 and multidrug resistant Dd2 strains of *Plasmodium falciparum* using a SyBr Green fluorescence-based assay. The cytotoxicity of active extracts, fractions, and compounds was tested against mammalian Raw cell lines using an in vitro resazurin-based viability assay. The structures of the compounds were determined based on their NMR and MS data. The in vivo toxicity using female BALB/c mice was performed on the most active extract according to the protocol of OECD (2002), guideline 423.

**Results:**

The hydroethanolic extract from the leaves of *D. edulis* displayed good antiplasmodial activity with IC_50_ values of 3.10 and 3.56 μg/mL respectively on sensitive (3D7) and multiresistant (Dd2) strains of *P. falciparum*. Of the sixteen compounds isolated, 3,3′,4-tri-*O*-methylellagic acid (**4**) exhibited the highest antiplasmodial activity against *Pf*Dd2 strains with an IC_50_ value of 0.63 μg/mL. All extracts, fractions, and isolated compounds demonstrated no cytotoxicity against Raw cell lines with CC_50_ > 250 μg/mL. In addition, the most active extract on both strains of *P. falciparum* was nontoxic in vivo, with a LD_50_ greater than 2000 and 5000 mg/kg. A phytochemical investigation of the stem bark and leaves of *D. edulis* afforded sixteen compounds, including two xanthones (1–2), three ellagic acid derivatives (3–5), one phenolic compound (**6**), one depside (**7**), one triglyceride (**8**), one auranthiamide acetate (**9**), one gallic acid derivative (**10**), four triterpenoids (**11**–**14**), and two steroids (**15**–**16**). Compounds **1**, **2**, **5**, **7**, **8**, and **9** were herein reported for the first time from the Burseraceae family.

**Conclusion:**

This work highlights the good in vitro antiplasmodial potency of the hydroethanolic extract of the leaves of this plant and that of two isolated constituents (3,3′,4-tri-*O*-methylellagic acid and ethylgallate) from the plant. These biological results support the use of *D. edulis* in traditional medicine against malaria.

**Supplementary Information:**

The online version contains supplementary material available at 10.1186/s12906-023-03957-2.

## Background

Emerging and/or re-emerging epidemic diseases represent an ongoing threat to global security [1]. The current situation that the whole world is experiencing with COVID-19 sufficiently illustrates this allegation. Despite the emergence of COVID-19, malaria still remains a major public health concern worldwide, with the African region being disproportionately affected [2]. According to the WHO (2021), among the approximately 1.5 million lives claimed each year by malaria, 96% of deaths occurred in Africa, with children under 5 years of age accounting for about 80% of all deaths [3]. The ravages caused by this disease are felt much more acutely in the developing countries due to the high costs of conventional drugs used in therapy, added to their precarious accessibility first, and second, to the increase in resistance that vectors and parasites developed toward the available medicines. Despite scientific efforts to control the pathogenic agent, *Plasmodium falciparum*, malaria continues to decimate the population mostly in Africa. Faced with these health issues, the search for new active, safer and more efficient antimalarials with a novel mode of action to combat pathogenic agents and of almost zero toxicity is becoming imperative. To find such agents, medicinal plant-based drug discovery remains a hard-wearing solution. As such, medicinal plants always have a strong historical link with malaria treatment, exemplified by quinine and artemisinin isolated from *Cinchona* trees and *Artemisia annua*, respectively. Hence, investigating plants widely used in traditional medicine to treat malaria can provide new drugs with an innovative biological mode of action. *Dacryodes edulis* (G. Don) HJ. Lam. (Burseraceae) is traditionally used alone or in association with other plants in folk medicine to cure wounds, fever, headaches, and malaria [4, 5]. *D. edulis* is a fruit tree that grows naturally in the rainforests of the Gulf of Guinea and the Congo Basin. Its current range due to culture extends from Central Africa to Uganda in the east and south to Angola [6]. It is a plant largely used for its nutritional and medicinal values and locally called bush butter tree and «safoutier» by English and French speaking people, respectively [5]. Previous phytochemical investigations of the stem bark of *D. edulis* led to the identification of several classes of secondary metabolites including alkaloids, steroids/triterpenoids, phenols, reducing sugars, cardiac glycosides, flavonoids, saponins, tannins, and anthraquinones [7, 8]. Additionally, analgesic, anti-inflammatory, anti-allergic, anticancer, antimicrobial, and antiplasmodial activities were also reported [9]. Knowing that the phytochemical composition of plants varies dramatically according to their geographical location, country, day, time and place of collection combined with our continuous search for bioactive compounds from Cameroonian medicinal plants, we investigated the leaves and stem bark of *D. edulis* with an emphasis on the antiplasmodial and cytotoxic effects of the extracts, fractions, and isolated compounds.

## Methods

### General experimental procedures

Column chromatography separations were performed on 230–400 mesh silica gel (Merck, Darmstadt, Germany), and Sephadex LH-20 (Sigma-Aldrich, Munich, Germany). Fractions were grouped using thin-layer chromatography (TLC) profiles with Merck precoated silica gel sheets (60 F_254_), and the identification of spots on the TLC plates was carried out by spraying with a solution of dilute sulfuric acid and heating the plate at approximately 80ºC. Compounds were visualized under UV light at 254 nm or 365 nm. The 1D and 2D NMR spectra were recorded on Bruker DRX 500 MHz and 600 MHz (Bruker, Rheinstetten, Germany) for ^1^H-NMR and 125 MHz and 150 MHz for ^13^C-NMR spectrometers, giving the chemical shifts in ppm and the coupling constants in Hertz.

### Plant material

The leaves and stem bark of *D. edulis* were collected in April 2017 in Batcham, West Region of Cameroon. Although, no specific license is required for *D. edulis*, its parts were collected upon oral approval by local authorities and in strict respect of the Cameroonian biodiversity rights and regulations. The plant material was identified by Mr. NANA Victor, botanist at the National Herbarium of Cameroon, Yaoundé by comparison with voucher specimens formally kept at the National Herbarium under the registration number 45713 HNC.

### Extraction and isolation

The air-dried and powdered stem bark (4.5 kg) of *D. edulis* was extracted with MeOH (15L × 4) (3 days, repeated two times) at room temperature (approximately 27℃). The extract was freed from the solvent under vacuum at low temperature (40℃) to give 239.8 g of crude extract. The crude extract was subjected to liquid–liquid partition using *n*-hexane, dichloromethane, EtOAc and *n*-BuOH to afford four fractions, including the *n*-hexane fraction (DEH) (20.5 g), dichloromethane fraction (DEC) (3.92 g), ethyl acetate fraction (DEA) (35.5 g), and *n*-butanol fraction (DEN) (43.2 g). The *n*-hexane fraction was subjected to flash column chromatography using silica gel (Merck, 230–400 mesh) eluting with *n*-hexane, a mixture of *n*-hexane-DCM gradient (1:0–0:1), a mixture of DCM-EtOAc (1:0–0:1), and EtOAc–MeOH (1:0–0:1) of increasing polarity to yield four main subfractions labeled A (4.7 g), B (3.8 g), C (5.3 g) and D (5.1 g). Subfraction A was subjected to CC over silica gel and eluted with an *n*-hexane/DCM mixture (1:0–0:1) to yield *β*-amyrin acetate (11) (5.7 mg) and *β*-amyrin (12) (8.4 mg). Subfraction B was also subjected to CC over silica gel and eluted with an *n*-hexane/DCM mixture (1:0–0:1) to yield griseoxanthone C (2) (9.4 mg) and a (1:1) mixture of *β*-and *α*- amyrin (14) (50.9 mg). Subfraction C was further submitted to CC over silica gel and eluted with *n*-hexane/DCM (1:0–0:1) to afford masticadienonic acid or 3-oxo-lanosta-7,24-*Z*-dien-26-oid acid (13) (10.5 mg) and confluentic acid (7) (6.0 mg). Subfraction D was subjected to CC over silica gel and eluted with an *n*-hexane/DCM mixture (1:0–0:1) to produce a (1:1) mixture of *β*-sitosterol and stigmasterol (15).

The DCM fraction was subjected to CC over silica gel (Merck, 230–400 mesh) and eluted with a mixture of *n*-hexane/EtOAc (1:0–0:1) and an EtOAc/MeOH mixture (1:0–0:1) of increasing polarity to afford lichexanthone (1) (3.1 mg), 3,3′-*O*-dimethylellagic acid (3) (3.7 mg), and *β*-sitosterol-3-*O*-*β*-D-glucopyranoside (16) (30.2 mg).

The EtOAc fraction was also subjected to CC over silica gel (Merck, 230–400 mesh) and eluted with an *n*-hexane/EtOAc mixture (1:0–0:1) and an EtOAc/MeOH mixture (1:0–0:1) of increasing polarity. Subfractions (120) of 100 mL each were collected and combined according to their TLC and LC–MS profiles to afford four subfractions (A-D). Subfraction B (8.5 g) was subjected to silica gel column chromatography (CC) and eluted with DCM-MeOH mixture (1:0–0.8:0.2) to yield 3,3′,4-tri-*O*-methylellagic acid (4) (50.0 mg). Subfraction C (5.1 g) was also subjected to silica gel column chromatography (CC) and eluted with DCM-MeOH mixture (1:0–1:0) to produce 3,3′′-di-*O*-methylellargic acid 4-*O*-(3′′-galloyl)-*β-*D-xylopyranoside (5) (6.7 mg) and 3,4-dihydroxybenzoic acid (6) (6.5 mg).

**Fig. 1:**
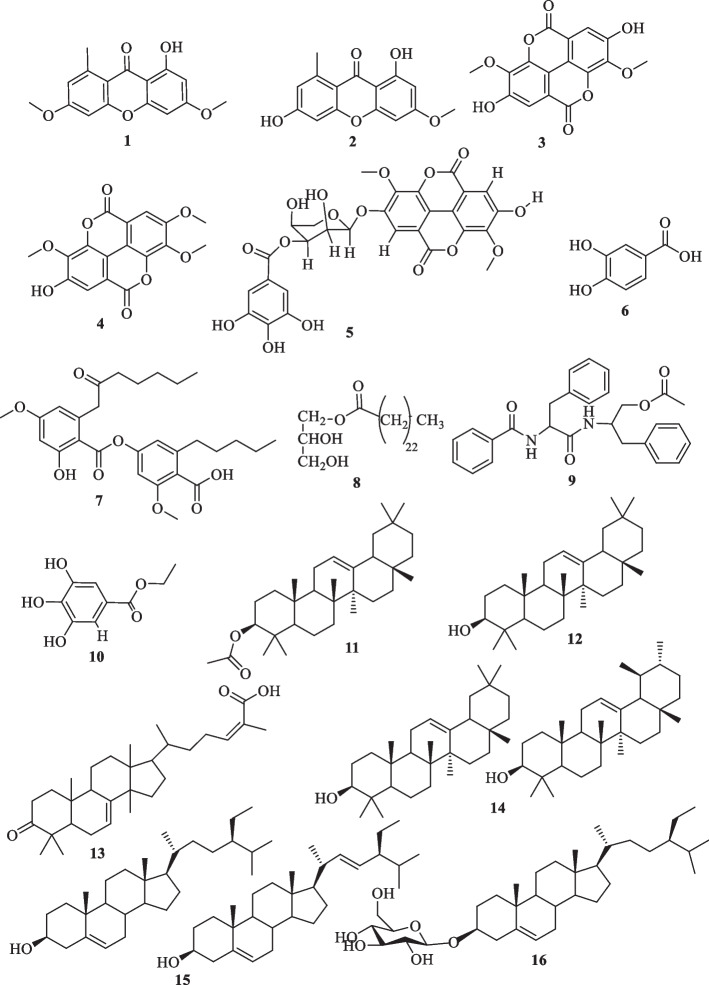
Structures of isolated compounds (**1**–**16**)

The air-dried and powdered leaves (2.5 kg) of *D. edulis* were extracted with a (7:3) mixture of ethanol–water (2.5 × 10 L) (three days, repeated two times) at room temperature (approximately 26℃). The extract was then freed from solvent under vacuum at low temperature (40℃) to give 205.5 g of crude extract. The crude extract was then subjected to liquid–liquid extraction with different solvents and gave four fractions including the *n*-hexane fraction (DFH) (25.7 g), dichloromethane fraction (DFC) (17.5 g), ethyl acetate fraction (DFA) (20.3 g), and *n*-butanol fraction (DFN) (102.3 g). The *n*-hexane fraction was subjected to flash column chromatography using silica gel (Merck, 230–400 mesh) eluting with mixture of *n*-hexane-DCM of increasing polarity to yield four compounds, *β*-amyrin acetate (11) [*n*-hexane/DCM (1:1), (8.3 mg)], *β*-and *α*- amyrin (14) [*n*-hexane/DCM mixture (1:1), (21.8 mg)], *β*-amyrin (12) [*n*-hexane/DCM (1:4), (4.1 mg)] and the mixture of *β*-sitosterol and stigmasterol (15) [DCM, (6.6 mg)]. The ethyl acetate fraction was assessed by CC over silica gel (Merck, 230–400 mesh) and eluted with a DCM/MeOH mixture (1:0–1:0) of increasing polarity to afford glyceryl-1-tetracosanoate (8), auranthiamide acetate (9) (4.9 mg), and ethyl gallate (10). The structures of these compounds (Fig. 1) were determined by comparison of their spectroscopic data with those reported in the literature.

## Bioassay

The in vitro and in vivo assays were performed at the Antimicrobial and Biocontrol Agents Unit, Laboratory of Phyto-biochemistry and Medicinal Plants Study, Department of Biochemistry, University of Yaoundé I, Cameroon. In vivo studies were performed in the strict respect of animal welfare and the data reported following the ARRIVE guidelines (Animal Research: Reporting of In Vivo Experiments) [10]. The collection of blood for *Plasmodium* parasite culture was achieved according to exclusion/inclusion criteria: Blood samples used for the study were collected from a subject who had not  received antimalarial drugs within the previous two months of blood collection and was free from malaria parasites following think blood smear.

### *Plasmodium falciparum* growth inhibition assay

#### In vitro* cultivation of P. falciparum*

The chloroquine-sensitive *Pf*3D7-(MRA-102) and chloroquine-resistant *Pf*Dd2*-*(MRA-150) *P. falciparum* strains were maintained as previously described [11] in fresh O^+^ human red blood cells at 4% haematocrit in complete RPMI 1640 medium [500 mL RPMI 1640 (Gibco, UK) supplemented with 25 mM HEPES (Gibco, UK), 50% albumax I (Gibco, USA), 1X hypoxanthine (Gibco, USA) and 50 mg/mL gentamicin (Gibco, China)] and incubating at 37 °C in a humidified atmosphere with 5% CO_2_. The medium was renewed daily to propagate the culture. Giemsa-stained thin blood smears were examined microscopically under immersion oil to monitor cell-cycle transition and parasitemia evolution. O^+^ human red blood cells were obtained from a consent volunteer donor (Supplementary material).

#### Blood specimen

Whole blood (25 mL) was drawn for a heathy volunteer without a historical contraceptive or anticoagulant therapy using a protocol approved by the Institutional Review Board (IRB No. 001/UY11 BTC/IRBI 2009), Biotechnology Centre, University of Yaoundé I, Cameroon. A consent form was approved by Fekam Boyom Fabrice, Professor and Head of the Antimicrobial & Biocontrol Agents Unit (AmBcAU), Laboratory for Phytobiochemistry and Medicinal Plants Studies, University of Yaoundé 1, Cameroon, for collection of blood sample from human volunteer.

#### Statement on informed consent of the donor

The volunteer donor was supplied a consent form which informed the purpose of the research project, name and details as well as contact person. Inclusion and exclusion criteria of the donor include volume of blood to be taken, age, sex, no medical illness, no medication, and time required for the blood sampling. Information’s were asked during face to face interview. Blood donor was aware that his sample might be used for research purpose and written consent form was obtained.

#### Synchronization of P. falciparum parasite culture

Before each experiment, synchronized ring-stage parasite were obtained by 5% sorbitol (w/v) treatment with respect to Lambros and Vanderberg, 1979 [12]. It is crucial to note that, using cultures over mixed stage cultures can enable the test molecules to interact with all three stages (ring, trophozoite, and schizont) of the 48 h long life cycle of *P. falciparum* in culture.

#### Antiplasmodial SYBR green I-based fluorescence assay

A drug sensitivity assay was carried out in 96-well microtitration plates using a SYBR green I-based fluorescence assay [13]. This assay is specifically based on the ability of SYBR green to give strong fluorescence only in the presence of parasite DNA during cell proliferation.

Shortly, sorbitol-synchronized ring stage parasites (hematocrit: 1%, parasitemia: 2%, 90 μl) under normal culture conditions were incubated in the presence of prediluted extracts, fractions, isolated compounds and reference drug (10 *μ*L) followed by incubation at 37℃ for 72 h. After incubation, 100 *μ*L of SYBR Green I buffer [6 *μ*L of 10,000 × SYBR Green I (Invitrogen) + 600 *μ*L of Red Blood Cells lysis buffer {Tris (25 mM; pH 7.5)} + 360 *μ*L of EDTA (7.5 mM) + 19,2 *μ*L of parasites lysis solution {saponin (0.012%; wt/vol)} and 28,8 *μ*L of Triton X-100 (0.08%; vol/vol)}] was added to each well, mixed twice gently with a multichannel pipette and incubated in the dark at 37℃ for 1 h [11]. Fluorescence was measured using a TECAN M 200 Microplate reader with excitation and emission at 485 and 538 nm, respectively. The fluorescence counts were plotted against the logarithm of the sample concentration and the 50% inhibitory concentration (IC_50_) was determined by analysis of dose–response curves using GraphPad Prism 5.0. Experiments were performed in duplicate.

#### In vitro* cytotoxicity assay*

The cytotoxicity profile of extracts, fractions and isolated compounds was assessed using the resazurin-based assay essentially as previously described [14] against RAW 264.7 cells duly cultivated in complete Dulbecco′s Modified Eagle′s Medium (DMEM) that contain 13.5 g/L of DMEM (Sigma Aldrich), 10% fetal bovine serum (Sigma Aldrich), 0.2% sodium bicarbonate (w/v) (Sigma Aldrich) and 50 μg/mL of gentamicin (Sigma Aldrich). Globally, macrophages (Raw cells) were seeded into 96-well cell-culture flat-bottomed plates at a density of 10^4^ cells in 100 *μ*L of complete medium/well and placed in incubation for 24 h at 37℃, and 5% CO_2_ allowing cell adhesion. Following cell adhesion, ten microliters of each serially diluted test sample solution was added to assay plates and left in incubation for 48 h under the same experimental conditions. Growth control (1% DMSO-100% growth) and positive control wells (podophyllotoxin at 20 μM) were included in the experimental plates. Cell proliferation was checked by adding 10 *μ*L of a stock solution of resazurin (0.15 mg/mL in sterile PBS) to each well followed by an additional incubation of 4 h in the same culture conditions. Fluorescence was then read using Tecan Infinite M200 fluorescence multiwell plate reader (Tecan) at an excitation/emission of 530/590 nm. The results were expressed as 50% cytotoxic concentrations (CC_50_) and selectivity indices (CC_50_ Mammalian cell/IC_50_ *Pf*3D7) were calculated for each test substance.

### In vivo acute toxicity

In vivo studies were performed in the animal house of the Laboratory of the Phyto-biochemistry and Medicinal Plants Study at the University of Yaoundé I, Cameroon, using female healthy BALB/c mice between 6—8 weeks old (20—25 g) maintained in constant and standard laboratory conditions (23—25℃ and light/dark cycles i.e. 12/12 h) with free access to food and tap water. Experiments with animals were performed according to the ethical standards formulated in the Declaration of Helsinki, and adequate measures were taken to protect animals from pain or discomfort, including sacrifice through injection of a mixture of ketamine (120 mg/kg) and xylazine (1 mg/kg). The protocol used for animal experimentation received approval from the Institutional Review Board (IRB No. 001/UY11 BTC/IRBI 2009), Biotechnology Centre, University of Yaoundé I, Cameroon.

To assess the safety of *D. edulis* in an animal model, healthy female *BALB/c* mice were randomly divided into three (03) groups of three (03) mice and treated by the oral route with a single dose of hydroethanolic leaf extract of *D. edulis* at doses of 2000 mg/kg and 5000 mg/kg while 20 mL/kg of distilled water was used for the control group. Oral gavage was chosen as a mode of administration to mimic the traditional route of administration as described previously [15]. Oral gavage was achieved as per the guidelines of the Organization for Economic Cooperation and Development (OECD) [16]. Following extract administration, animals were observed for 30 min and the first 4 h after treatment to record immediate deaths and once daily for 14 days to record any manifestation of toxicity.

## Results and discussion

### Isolated compounds

The methanol and hydroethanolic extracts from the stem bark and leaves of *D. edulis*, respectively, were subjected to silica gel and Sephadex LH-20 column chromatography (CC) to yield 16 known compounds (1–16) (Fig. 1). The structures of the isolates were determined from their spectroscopic data (UV, IR, NMR and MS) and by comparison with those of similar compounds reported in the literature.

Lichexanthone (1): white powder, HR-ESI–MS (positive mode, *m/z*): 287.0935 [M + H]^+^, (calcd 287.0914 for C_16_H_15_O_5_). ^1^H-NMR (Pyridin-*d*_5_, 500 MHz): 2.91 (3H, s, -CH_3_), 3.78 (3H, d, *J* = 1.5 Hz, -OCH_3_), 3.81 (3H, d, *J* = 1.5 Hz, -OCH_3_), 6.55 (1H, d, *J* = 2.1 Hz, H-2), 6.60 (1H, d, *J* = 2.1 Hz, H-4), 6.79 (1H, d, *J* = 2.4 Hz, H-5), 6.86 (1H, d, *J* = 2.2 Hz, H-7), 13.91 (1H, -OH). ^13^C-NMR (Pyridin-*d*_5_, 125 MHz): 23.8 (C-15), 56.2 (C-16), 56.2 (C-14), 92.8 (C-4), 97.8 (C-2), 99.5 (C-5), 104.7 (C-10), 113.4 (C-13), 116.3 (C-7), 143.8 (C-8), 157.7 (C-11), 160.0 (C-12), 164.7 (C-6), 164.8 (C-1), 166.8 (C-3), 183.0 (C-9) [16].

Griseoxanthone C (2): white powder, HR-ESI–MS (positive mode, *m/z*): 274.0837 for [M + 2H]^+^, (calcd 274.0836 for C_15_H_14_O_5_).^1^H-NMR (CDCl_3_, 600 MHz): 13.39 (1H, OH), 6.68 (1H, s, H-7), 6.66 (1H, s, H-5), 6.35 (1H, s, H-4), 6.31 (1H, s, H-2), 3.86 (3H, s, OCH_3_), 2.88 (3H m, -CH_3_). ^13^C-NMR (CDCl_3_, 150 MHz): 182.6 (C-9), 165.9 (C-3), 163.8 (C-1), 163.7 (C-6), 159.4 (C-11), 156.9 (C-12), 143.5(C-8), 115.6 (C-7), 113.0 (C-13), 104.2 (C-10), 98.5 (C-5), 96.8 (C-2), 92.1 (C-4), 55.7 (C-14), 23.6 (C-15) [17].

3,3′-*O*-Dimethylellargic acid (3): white powder, HR-ESI–MS (positive mode, *m/z*): 353.0268 for [M + Na]^+^, (calcd 353.0260 for C_16_H_10_O_8_Na). ^1^H-NMR (Pyridin-*d*_5_, 500 MHz): 3.59 (3H, s, -OCH_3_), 4.17 (3H, s, -OCH_3_), 4.99 (1H, -OH), 8.04 (1H, s) [18].

3,3′,4-Tri-*O*-methylellagic acid (4): white powder, HR-ESI–MS (positive mode, *m/z*): 367.0425 for [M + Na]^+^. (calcd 367.0424 for C_17_H_12_O_8_Na). ^1^H-NMR (Pyridin-*d*_5_, 500 MHz): 3.85 (3H, s, -OCH_3_), 4.13 (3H, s, -OCH_3_), 4.19 (3H, s, -OCH_3_), 7.82 (1H, -OH), 8.03 (1H, s). ^13^C-NMR (Pyridin-*d*_5_, 125 MHz): 56.4 (-OCH_3_), 61.1 (-OCH_3_), 61.3 (-OCH_3_), 107.8 (C-5′), 111.6 (C-1′), 112.6 (C-1), 112.9 (C-5), 113.6 (C-6′), 114.1 (C-6), 141.1 (C-2), 142.1 (C-3′), 141.8 (C-2′), 142.1 (C-3), 154.1 (C-4′), 154.3 (C-4), 158.9 (C-7′), 159.0 (C-7) [18].

3,3′′-Di-*O*-methylellargic acid 4-*O*-(3′′-galloyl)-*β*-*D*-xylopyranoside (5): white powder, HR-ESI–MS (positive mode, *m/z*): 637.0839 for [M + Na]^+^, (calcd 637.0806 for C_28_H_22_O_16_Na). ^1^H-NMR (MeOD, 600 MHz): 8.66 (1H, s, H-5), 8.36 (1H, s, H-5′), 7.85 (2H, s, H-2′′ and H-6′′), 6.20 (2H, d, *J* = 7.4 Hz), 5.91 (2H, d, *J* = 9.3 Hz, H-1′′), 4.73 (1H, dd, *J* = 11.1; 5.2 Hz), 4.53 (1H, dq, *J* = 9.5, 4.4 Hz), 4.46 (1H, d, *J* = 6.9 Hz), 4.38 (1H, t, *J* = 10.7 Hz), 4.88 (3H, s, OCH_3_), 4.87 (3H, s, OCH_3_). ^13^C-NMR (MeOD, 150 MHz): 174.9 (C-7‴), 168.0 (C-7), 167.9 (C-7′), 162.5 (C-4′), 160.5 (C-4), 155.0 (C-3‴/5‴), 151.5 (C-6), 151.2 (C-6′), 150.5 (C-5), 149.7 (C-5′), 147.7 (C-4‴), 129.4 (C-1‴), 124.1 (C-2), 122.4 (C-2′), 122.6 (C-1′), 121.5 (C-1), 121.2 (C-3), 120.6 (C-3′), 118.3 (C-2‴), 118.3 (C-6‴), 110.9 (C-1″), 86.7 (C-3″), 80.7 (C-2″), 76.9 (C-4″), 75.2 (C-5″), 71.2 (OCH_3_), 70.5 (OCH_3_) [19].

3,4-Dihydroxybenzoic acid (6): white powder, ^1^H-NMR (MeOD, 600 MHz): 7.45 (1H, d, *J* = 2.0 Hz, H-2), 7.44 (1H, d, *J* = 2.1 Hz, H-6), 6.82 (1H, d, *J* = 7.9 Hz, H-3). ^13^C-NMR (MeOD, 150 MHz): 170.0 (CO), 151.3 (C-4), 145.9 (C-5), 123.6 (C-1), 122.9 (C-3), 117.5 (C-6), 115.5 (C-2) [20].

Confluentic acid (7): white powder, HR-ESI–MS (positive mode, *m/z*): 523.2336 for [M + Na] ^+^, (calcd 523.2302 for C_28_H_36_O_8_Na). ^1^H NMR (CDCl_3_, 600 MHz): 11.36 (OH), 6.62 (1H, d, *J* = 2.0 Hz, H-5′), 6.56 (1H, d, *J* = 2.1 Hz, H-3′′), 6.49 (1H, d, *J* = 2.6 Hz, H-3), 6.32 (1H, d, *J* = 2.6 Hz, H-5), 4.10 (2H, s, H-8), 2.75 (2H, m, H-8′′), 2.44 (2H, m, H-10), 1.65 (2H, m, H-9′), 1.55 (2H, m, H-11), 1.36 (4H, m, H-10′′ and H-11′′), 1.22 (2H, m, H-12 and H-13), 0.91 (3H, m, H-12′′), 0.85 (3H, t, *J* = 7.1 Hz, H-14). ^13^C-NMR (CDCl_3_, 150 MHz): 207.1 (C-9), 170.5 (C-7′′), 169.2 (C-7), 166.6 (C-2), 164.9 (C-4), 157,8 (C-2′′), 151.4 (C-4′′), 144.5 (C-6′′), 138.9 (C-6), 119.9 (C-1′′), 115.2 (C-5′′), 113.4 (C-5), 104.2 (C-1), 103.1 (C-3′′), 100.1 (C-3), 56.4 (-OCH_3_), 55.5 (-OCH_3_), 51.2 (C-8), 42.6 (C-10), 33.8 (C-8′′), 31.6 (C-10′′), 31.3 (C-12), 30.7 (C-9′′), 23.4 (C-11), 22.5 (C-11′′), 22.4 (C-13), 14.0 (C-14), 13.9 (C-12′′) [21].

Glyceryl-1-tetracosanoate (8): white Powder, HR-ESI–MS (positive mode, *m/z*): 465.4543 for [M + Na]^+^.^1^H-NMR (CDCl_3_, 500 MHz): 4.21 (1H, dd, *J* = 11.7, 4.5 Hz, H-1), 4.15 (1Hb, dd, *J* = 11.7, 6.2 Hz, H-1), 3.93 (1H, tt, *J* = 6.0, 4.2 Hz, H-2), 3.69 (1Ha, dd, *J* = 11.4, 4.0 Hz, H-3), 3.60 (1H, dd, *J* = 11.4, 5.8 Hz, H-3), 2.35 (2H, t, *J* = 7.6 Hz, H-2′), 1.63 (2H, q, *J* = 7.5 Hz, H-3ʹ), 1.25 (nH, brs, H-4ʹ/H-23ʹ), 0.88 (3H, t, *J* = 7.0 Hz, H-24ʹ). ^13^C-NMR (CDCl_3_, 125 MHz): 174.4 (C-1ʹ), 70.2 (C-2), 65.2 (C-1), 63.6 (C-3), 34.3 (C-2ʹ), 24.9 (C-3ʹ), 29.7- 32.0 (C4ʹ-23ʹ), 14.1 (C-24ʹ) [22].

Auranthiamide acetate (9): white powder, HR-ESI–MS (positive mode, *m/z*): 455.2156 for [M + H]^+^, (calcd 523.2122 for C_27_H_29_O_4_N_2_). ^1^H-NMR (CDCl_3_, 500 MHz): 7.71 (1H, s, H-3), 7.52 (1H, s, H-3), 7.44 (2H, dddd, *J* = 8.5, 6.7, 1.6, 1.0 Hz, H-3), 7.25 (1H, d, *J* = 3.2 Hz, H-3), 7.17 (2H, ddt, *J* = 8.0, 6.6, 1.3 Hz, H-3), 6.71 (1H, d, *J* = 7.6 Hz, H-3), 5.90 (1H, d, *J* = 8.6 Hz, H-3), 3.93 (1H, dd, *J* = 11.3, 4.9 Hz, H-1), 3.82 (1H, dd, *J* = 11.3, 4.2 Hz, H-3), 3.22 (1H, dd, *J* = 13.7, 5.9 Hz, H-3), 3.05 (1H, dd, *J* = 13.7, 8.5 Hz, H-3). ^13^C-NMR (CDCl_3_, 125 MHz): 170.7 (C-2), 170.2 (C-6), 167.1 (C-9), 136.7 (C-1ʹ), 136.6 (C-1″), 132.0 (C-1‴), 129.3 (C-4‴), 129.1 (C-4ʹ), 129.1 (C-3‴/5‴), 128.7 (C-2‴/6‴), 128.6 (C-3″/5″), 128.5 (C-2ʹ/6′), 127.1 (C-2″/6″), 127.0 (C-3ʹ/5′), 126.7 (C-4″), 64.5 (C-3), 55.1 (C-7), 49.4 (C-4), 38.3 (C-11), 37.4 (C-10), 20.8 (C-1ʺ) [23].

Ethyl gallate (10): white powder, ^1^H-NMR (MeOD, 600 MHz): 7.06 (2H, s), 4.29 (2H, q, *J* = 7.1 Hz), 1.36 (3H, t, *J* = 7.1 Hz). ^13^C-NMR (MeOD, 150 MHz): 167.1(CO), 145.1 (C-3,5), 138.3 (C-4), 120.2 (C-1), 108.4 (C-2,6), 60.2 (CH_2_), 13.0 (CH_3_) [20].

*β*-Amyrinacetate (11): white powder.^1^H-NMR (CDCl_3_, 600 MHz,): 5.20 (1H, t, *J* = 3.7 Hz,), 4.55- 4.50 (1H, m), 2.07 (7H, s), 1.60 (2H, s), 1.15 (3H, s), 0.98 (2 × 3H, d, *J* = 2.9 Hz), 0.90–0.88 (4 × 3H, m), 0.85 (2H, s). ^13^C-NMR (CDCl_3_, 150 MHz,): 171.4 (CO), 145.8 (C-13), 121.8 (C-12), 80.9 (C-3), 55.3 (C-5), 47.6 (C-9), 47.2 (C-18), 46.8 (C-19), 41.7 (C-14), 39.8 (C-8), 38.3 (C-1), 37.7 (C-4), 37.1 (C-22), 36.8 (C-10), 34.8 (C-21), 33.5 (C-17), 32.6 (C-7), 31.2 (C-20), 28.4 (C-28), 28.1 (C-23), 26.9 (C-2), 26.1 (C-15), 26.0 (C-16), 25.9 (C-27), 23.7 (C-11), 23.6 (C-29), 23.5 (C-30), 21.4 (CH_3_CO), 18.3 (C-6), 16.8 (C-24), 16.7 (C-25), 15.6 (C-26) [24].

*β*-Amyrin (12): white powder, ^1^H-NMR (CDCl_3_, 600 MHz): 5.63 (1H, dd, *J* = 5.2, 2.9 Hz), 3.46 (1H, dd, *J* = 3.5, 2.3 Hz), 1.27- 1.23 (3H, m), 1.15 (6H, d, *J* = 10.6 Hz), 1.09 (3H, s), 1.04 (3H, s), 1.00 (6H, d, *J* = 7.8 Hz), 0.95 (3H, s), 0.85 (3H, s). ^13^C-NMR (CDCl_3_, 150 MHz): 141.7 (C-13), 122.0 (C-12), 76.4 (C-3), 49.7 (C-5), 47.5 (C-9), 43.1 (C-18), 40.9 (C-19), 39.3 (C-14), 38.9 (C-8), 37.8 (C-1), 36.1 (C-4), 35.1 (C-22), 34.8 (C-10), 34.6 (C-21), 34.5 (C-17), 33.1 (C-7), 32.4 (C-20), 32.1 (C-28), 32.0 (C-23), 30.4 (C-2), 30.1 (C-15), 28.9 (C-16), 28.3 (C-27), 27.8 (C-11), 25.5 (C-29), 23.6 (C-30), 19.6 (C-6), 18.4 (C-24), 18.2 (C-25), 16.2 (C-26) [24].

Masticadienonic acid or 3-oxo-lanosta-7,24-*Z*-dien-26-oid acid (13): white powder, HR-ESI–MS (positive mode, *m/z*): 455.3526 for [M + H]^+^. ^1^H-NMR (CDCl_3_, 600 MHz): 6.93 (1H, m, H-24), 5.67 (1H, m, H-7), 2.52 (1H, m, H-2), 0.93 (1H, dd, *J* = 6.4, 1.6 Hz, H-27), 0.83 (1H, d, *J* = 1.6 Hz, H-22). ^13^C-NMR (CDCl_3_, 150 MHz): 219.1 (C-3), 173.2 (C-26), 148.9 (C-8), 145.5 (C-24), 126.6 (C-25), 121.2 (C-7), 52.9 (C-17), 52.3 (C-5), 52.0 (C-14), 47.0 (C-4), 45.4 (C-9), 44.0 (C-13), 34.2 (C-2), 18.2 (C-27) [25].

### Antiplasmodial activity screening

**Fig. 2:**
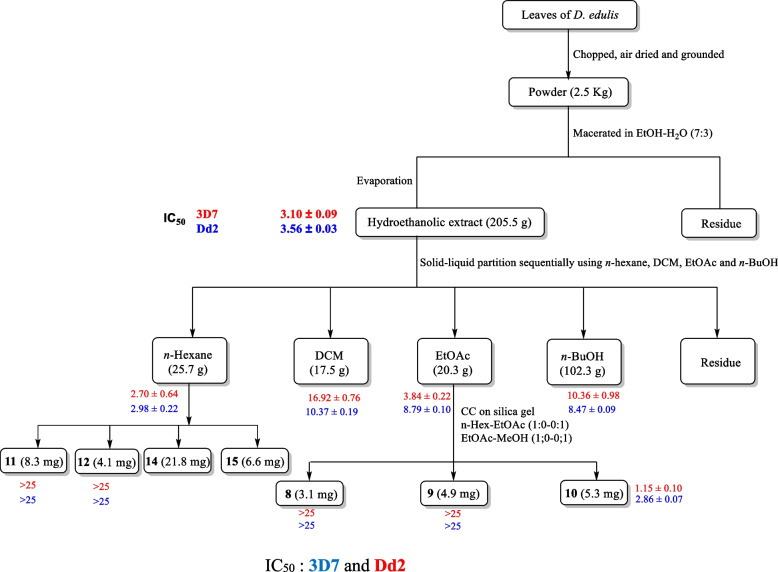
Bio-assay guided isolation of active compounds from the hydroethanolic crude extract of leaf of *D. edulis*

**Fig. 3:**
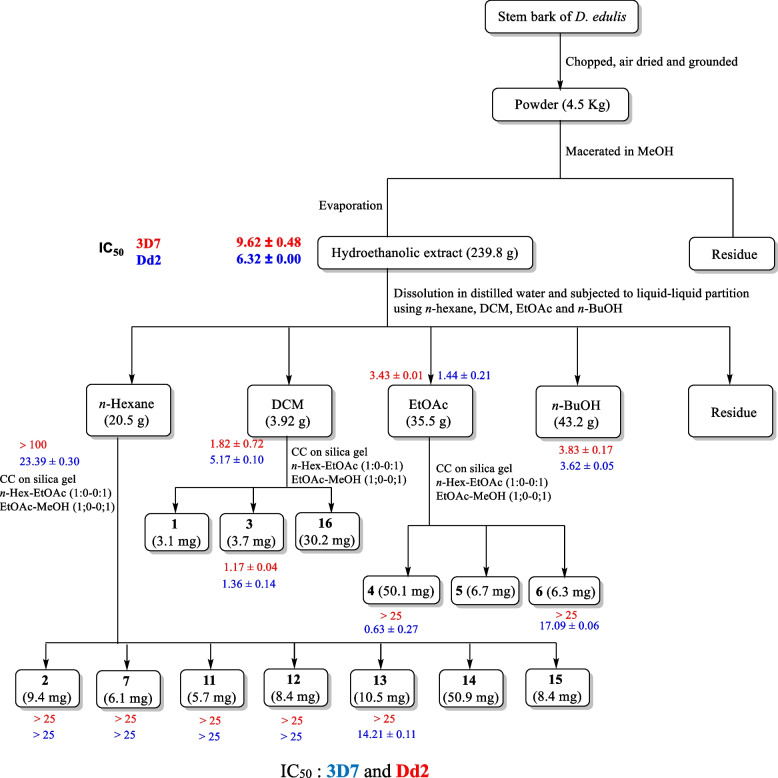
Bio-assay guided isolation of active compounds from the methanol crude extract of the stem bark of *D. edulis*

The *in-vitro* antiplasmodial activity screening of the methanol and hydroethanolic extracts, fractions and isolates of the leaves and stem bark of *D. edulis* against the chloroquine-sensitive (3D7) and the multidrug-resistant (Dd2) strains of *P. falciparum* was evaluated by measuring growth inhibition based on SYBR green fluorescence. Chloroquine (CQ) and artemisinin were used as reference compounds. To classify the antiplasmodial activity expressed as IC_50_ of tested samples, the following criteria were adopted: IC_50_ ≤ 5 μg/mL: pronounced activity; 5 < IC_50_ ≤ 10 μg/mL: good activity; 10 < IC_50_ ≤ 20 μg/mL: moderate activity; 20 < IC_50_ ≤ 40 μg/mL: low activity; IC_50_ > 40 μg/mL: inactive [26]. According to these criteria and as shown in Table 1, both methanol and hydroethanolic extracts and fractions from the leaves and stem bark of *D. edulis* inhibited the growth of both the *P. falciparum* 3D7 and Dd2 strains with IC_50_ values ranging from 1.44 to 23.39 μg/mL (Fig. 2). The methanol extract from the stem bark displayed good antiplasmodial activity against both *Pf3D7* and *PfDd2*, with IC_50_ values of 9.62 and 6.32 μg/mL, respectively. The hydroethanolic leaf extract of *D. edulis* exhibited pronounced antiplasmodial activity with IC_50_ values of 3.10 and 3.56 μg/mL, respectively against both the *Pf*_3D7_ and *Pf*_Dd2_ strains. Interestingly, the hydroethanolic leaf extract was more potent than the methanol stem bark extract. The difference observed in antiplasmodial activities of both extracts could be attributed to the chemical composition of the extracts or the part of the plant used. Additionally, four fractions obtained from the methanol stem bark extract and five fractions obtained from the hydroethanol leaf extract of *D. edulis* also exhibited good inhibition of *P. falciparum* 3D7 and Dd2 with IC_50_ values ranging from 1.44 to 23.39 μg/mL. Fraction DEA and DEN from the methanol stem bark extract of *D. edulis* exhibited pronounced antiplasmodial activity on both sensitive and resistant strains (DEA: IC_50_ *Pf*_3D7_: 3.43 and IC_50_ *Pf*_Dd2_:1.44 μg/mL; DEN: IC_50_ *Pf*_3D7_: 3.83 and IC_50_ *Pf*_Dd2_: 3.62 μg/mL) (Fig. 3).

**Table 1:** Results of antiplasmodial screening against *Pf3D7* and *PfDd2* and selectivity on Raw cell lines

	**SYBr Green based assay**			**Cytotoxicity assay on Raw Cells**		
	**IC50 ± SD (*μ*g/mL)**		**Resistance Index (RI)**	**CC**_**50**_** (*μ*g/mL)**	**SI**	
	***Pf*3D7**	***Pf*Dd2**			***Pf*3D7**	***Pf*Dd2**
**Extracts and fractions**						
**DEM**	9.62 ± 0.48	6.32 ± 0.00	0.65	> 250	> 26	> 40
**DEF**	3.10 ± 0.09	3.56 ± 0.03	1.14	> 250	> 81	> 70
**DEH**	> 100	23.39 ± 0.30	-	> 250	-	> 11
**DEC**	1.82 ± 0.72	5.17 ± 0.10	2.84	> 250	> 137	> 48
**DEA**	3.43 ± 0.01	1.44 ± 0.21	0.41	> 250	> 73	> 174
**DEN**	3.83 ± 0.17	3.62 ± 0.05	0.94	ND	-	-
**DFH**	2.70 ± 0.64	2.98 ± 0.22	1.31	> 250	> 93	> 84
**DFC**	16.92 ± 0.76	10.37 ± 0.19	0.61	> 250	> 15	> 24
**DFA**	3.84 ± 0.22	8.79 ± 0.10	2.28	> 250	65	> 28
**DFN**	10.36 ± 0.98	8.47 ± 0.09	0.81	> 250	> 24	> 30
**DFM**	8.72 ± 1.28	4.56 ± 0.19	0.52	> 250	> 29	> 55
**Compounds**						
**Griseoxanthone C (2)**	> 25	> 25	-	> 100	-	-
**3,3'-di-*O*-methylellargic acid (3)**	1.17 ± 0.04	1.36 ± 0.14	1.16	> 100	> 85	> 74
**3,3′,4-tri-*O*-methylellagic acid (4)**	> 25	0.63 ± 0.27	-	> 100	-	159
**3,3″-di-*O*-methylellagic acid 4-*O*-(3″-galloyl)-*β*-D-xylopyranoside (5)**	1.86 ± 0.18	1.76 ± 0.14	1.12	> 100	> 87	> 64
**3,4-dihydroxybenzoic acid (6)**	> 25	17.09 ± 0.06	-	> 100	-	> 6
**Confluentic acid (7)**	> 25	> 25	-	> 100	-	-
**Glyceryl-1-tetracosanoate (8)**	> 25	> 25	-	> 100	-	-
**Auranthiamide acetate (9)**	> 25	> 25	-	> 100	-	-
**Ethyl gallate (10)**	1.15 ± 0.10	2.86 ± 0.07	2.48	> 100	> 87	> 35
***β*-amyrinacetate (11)**	> 25	> 25	-	> 100	-	-
***β*-amyrin (12)**	> 25	> 25	-	ND	-	-
**3-oxo-lanosta-7,24-*Z*-dien-26-oid acid (13)**	> 25	14.21 ± 0.11	-	> 100	-	> 7
**Mixture of *β*- and *α*- amyrin (14)**	> 25	3.14 ± 0.09	-	> 100	-	> 32
**Artemisinin (nM)**	14.66 ± 0.11	18.90 ± 0.13	0.12	-	-	-
**Chloroquine (nM)**	4.36 ± 0.53	133 ± 0.16	30.5	-	-	-

*DEM* Methanolic extract of the stem bark of *D. edulis*, *DEF* Hydroethanolic extract of the leaves of *D. edulis*, *DEH* *n*-Hexane fraction of DEM, *DEC* DCM fraction of DEM, *DEA* AcOEt fraction of DEM, *DEN n*-BuOH fraction of DEM, *DFH* *n*-Hexane fraction of DEF, *DFC* DCM fraction of DEF, *DFA* AcOEt fraction of DEF, *DFN n*-BuOH fraction of DEF, *DFM* methanolic fraction of DEF, *IC*_*50*_ Inhibitory concentration 50%, *SI* Selectivity index, *CC*_*50*_ Cytotoxic Concentration 50, *Pf* *Plasmodium falciparum*

Among fractions from the hydroethanol leaf extract, fraction DFH was the only one showing pronounced antiplasmodial activity on both strains (IC_50_ *Pf*_3D7_: 2.70 and IC_50_ *Pf*_Dd2_: 2.98 μg/mL). Overall, we can observe that fractionation of both extracts led to some fractions with more potent and pronounced activities than the mother extracts. This observation could be explained by the fact that the phytochemical constituents responsible for the antiplasmodial activity in the mother extract were concentrated in potent fractions during the fractionation process. Of note, fractionation of an extract can positively or negatively change its biological properties by concentrating active ingredients into a fraction, or by sharing them between the various fractions [27]. Among the compounds isolated from both extracts, 3,3′-*O*-dimethylellagic acid (3) and ethylgallate (10) displayed pronounced antiplasmodial activities on both *Pf*_3D7_ and *Pf*_Dd2_. Interestingly, 3-oxo-7,24-*Z*-tirucalladien-26-oic acid (13); a (1:1) mixture of *β*- and *α*- amyrin (14), 3,3′,4-tri-*O*-methylellagic acid (4) and 3,4-dihydroxybenzoic acid (6) exhibited pronounced to moderate antiplasmodial activity only on the multidrug-resistant Dd2 strain of *P. falciparum* with IC_50_ values ranging from 0.63 to 17.09 μg/mL. However, 3, 3′, 4-tri-*O*-methylellagic acid (4) and the mixture of *α*- and *β*- amyrin (14) were the most potent compounds with respective IC_50_ values of 3.14 and 0.63 μg/mL, whereas 3,4-dihydroxybenzoic acid (6) and 3-oxo-lanosta-7,24-*Z*-dien-26-oid acid (13) displayed moderate antiplasmodial activity (Fig. 3). Overall, two hit compounds were identified among the isolates; one from the methanol stem bark extract (3, 3′-*O*-dimethylellargic acid) (3) and the other from the hydroethanolic leaf extract (ethyl gallate) (10) with very good antiplasmodial potency. These isolates could be interesting starting materials for further structure–activity relationship studies. The results summary is shown in Table 1 below.

**Fig. 4:**
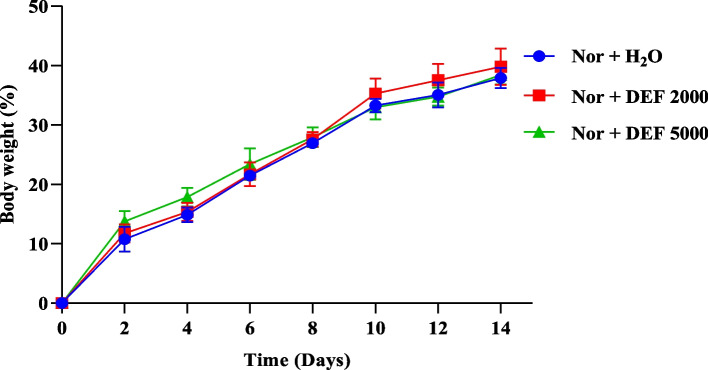
Effects of the aqueous extract of *D. edulis* leaf (DEF) on weight development in acutely toxic BALB/C mice. Legend: Nor: Normal; DEF: hydroethanolic extract of the leaves of *D. edulis* Nor + H_2_O: Normal mice receiving distilled water; Nor + DEF 2000: Normal mice receiving DEL extract at 2000 mg/kg; Nor + 5000: Normal mice receiving DEL extract at 5000 mg/kg

*In* vitro cytotoxicity profiles showed that all active extracts, fractions and isolated compounds were nontoxic with half maximal cytotoxic concentrations (IC_50_) values > 250 μg/mL for the tested extract and all the fractions and > 100 μg/mL for the isolated compounds. Futhermore, the hydroethanolic extract of *D. edulis* leaf extract at the tested limit doses of 2000 and 5000 mg/kg did not cause any death during the 14 days of experimentation, indicating that the median lethal dose (LD_5__0_) of this extract is greater than 5000 mg/kg. Therefore, according to the OECD’s Globally Harmonized System of Classification [16], *D. edulis* leaf extract can be classified as category 5 and considered nontoxic once administered orally. Additionally, the recording of body weight during 14 days of observation showed no significant change in treated animals compared to the normal group (Fig. 4). Combining above mentioned good results in both in vitro cytotoxicity and in vivo acute toxicity tests, *D. edulis* is a source of nontoxic molecules suitable for further investigation toward the search for new antimalarial drugs.

The antiplasmodial activity of the methylene chloride/methanol extract of *D. edulis* have already been reported by Zofou and colleagues (2013) who found significant activity against both chloroquine-sensitive (3D7) and chloroquine-resistant (Dd2) strains of *P. falciparum* with IC_50_ values of 4.34 and 6.43 μg/mL, respectively [9]. In 2022, Vegara and collaborators reported the antiplasmodial activity of the ethanol extract from the bark of *Bursera simaruba* (Burseraceae) from Colombian North Coast with IC_50_ values of 1.7 and 1.2 μg/mL on the chloroquine sensitive (3D7) and chloroquine-resistant (Dd2) strains, respectively [28]. However, our study demonstrates the antiplasmodial activities of the methanol stem bark extract and hydroethanol leaf extract and underscores the fact that regardless of the solvent or the part of the plant used, *D. edulis* constitutes a powerful source of antiplasmodial compounds. Nevertheless, in the work conducted by Zofou and colleagues, fractionation of methylene chloride/methanol extract led to the isolation of quercitrin, afzelin, quercetin, methyl 3,4,5-trihydroxybenzoate, and sitosterol 3-*O*-*β-D*-glucopyranoside sterol with antiplasmodial activities ranging from 0.37 to 18.53 μg/mL against both sensitive *Pf*3D7 and resistant *Pf*Dd2 strains of *P. falciparum*. This discrepancy in activities could be explained by the different extraction procedures, the part of plant used, which is directly linked to the quantity and quality of secondary metabolites present in the crude extract [29]. Besides, 3,4-dihydroxybenzoic acid (6) isolated in this study showed moderate activity (IC_50_: 17.09 μg/mL) on multi-resistant (Dd2) strain of *P. falciparum* compared to methyl 3,4,5-trihydroxybenzoate (DES4) (IC_50_: 0.56 μg/mL) isolated from the work achieved by Zofou and co-workers in 2013. The activity obtained by Zofou et al. is 30.51-fold more potent than the one obtained in our study. This discrepancy could be explained by the hydroxyl group at position 5 in methyl 3,4,5-trihydroxybenzoate absent in methyl 3,4-trihydroxybenzoate. From our investigation, 3,3'-di-*O*-methylellagic acid (3), 3,3″-di-*O*-methylellagic acid 4-*O*-(3″-galloyl)-*β*-D-xylopyranoside (5), Ethyl gallate (10) 3,3′,4-tri-*O*-methylellagic acid (4) belonging to the class of phenolics compound and derivative have been pointing out as the most highly potent antiplasmodial agents from *D. Eludis*. Phenolics compounds and derivative have been reported to displayed strong antiplasmodial potency. Of note, phenolic compounds are frequent in plants and are distributed within numerous phytochemical classes. They are broadly defined by their molecular structure, with one or more aromatic rings, without a nitrogen, originated from plantʼs metabolism pathways of the shikimate and/or acetate [30]. Some phenolic compounds such as ellagic acid have been reported to have strong antiplasmodial activity across sensitive and resistant strains of *P. falciparum* by interfering with DNA topoisomerase, the induction of cell cycle arrest, and the activation of apoptotic pathways [31]. Besides, its mechanism of action is also attributed to the inhibition of Plasmepsin II, the reduction of glutathione content inside the *Plasmodium* parasite and an impairment of beta-hematin formation [31–33]. Although the mechanism of action ethyl gallate on *P. falciparum* have not yet been elucidated, reported data suggests that the hydroxy groups of the gallate moiety could play a pivotal role as donors in the establishment of bonds where the compound would exercise its activity [30]. The finding from this investigation showed that *D. eludis* is an inexhaustible source of drug candidate with antimalarial properties.

## Conclusion

The objective of this work were to investigate the antiplasmodial activities and the toxicity of *D. edulis* extracts from the leaves and the stem bark as well as their fractions and isolated compounds. The hydroethanolic leaves displayed better antiplasmodial activity than the methanolic extract of the stem bark, with IC_50_ values of 3.10 and 3.56 μg/mL on 3D7 and Dd2*,* respectively. Among the sixteen compounds isolated, 3,3′,4-tri-*O*-methylellagic acid exhibited the highest antiplasmodial activity against *Pf*Dd2 strains with an IC_50_ value of 0.63 μg/mL. All extracts, fractions, and isolated compounds demonstrated cytotoxicity against Raw cell lines with CC_50_ > 250 μg/mL. Moreover, the most active extract on both strains of *P. falciparum* was nontoxic in vivo with an LD_50_ greater than 5000 mg/kg. The results obtained in the present work highlighted safety and the antiplasmodial potency of the hydroethanolic extract of the leaves of this plant as well as the antiplasmodial activity of 3,3′,4-tri-*O*-methylellagic acid and ethylgallate. These biological results justify the use of *D. edulis* in traditional medicine against malaria, and can therefore contribute to hit optimization studies in antimalarial drug development .

## Supplementary Information

**Additional file 1:** **Figure S1. **HR-ESI mass spectrum of 1.** Figure S2. **1H NMR spectrum(Pyridin-*d5*, 600 MHz) of 1.** Figure S3. **13C NMR spectrum (Pyridin-*d5*, 150 MHz) of 1. **Figure S4. **ESI mass spectrum of2. **Figure S5. **1H NMR spectrum (CDCl3, 600 MHz) of 2. **Figure S6. **13C NMR spectrum (CDCl3, 150 MHz) of 2. **Figure S7. **1H NMR spectrum(Pyridin-*d5*, 600 MHz) of 3. **Figure S8. **1H NMR spectrum(Pyridin-*d5*, 600 MHz) of 4. **Figure S9. **13C NMR spectrum(Pyridin-*d5*, 150 MHz) of 4. **Figure S10. **HR-ESI mass spectrum of 5. **Figure S11. **1H NMRspectrum (CD3OD, 600 MHz) of 5. **Figure S12. **13C NMRspectrum (CD3OD, 150 MHz) of 5. **Figure S13. **1H NMRspectrum (CD3OD, 600 MHz) of 6. **Figure S14. **13C NMRspectrum (CD3OD, 150 MHz) of 6. **Figure S15. **HR-ESI mass spectrum of 7.** Figure S16. **1H NMRspectrum (CDCl3, 600 MHz) of 7.** Figure S17. **13C NMRspectrum (CDCl3, 150 MHz) of 7.** Figure S18. **1H NMRspectrum (CDCl3, 600 MHz) of 8. **Figure S19. **13C NMRspectrum (CDCl3, 150 MHz) of 8. **Figure S20. **HR-ESI mass spectrum of 9. **Figure S21. **1H NMRspectrum (CDCl3, 600 MHz) of 9. **Figure S22. **13C NMRspectrum (CDCl3, 150 MHz) of 9. **Figure S23. **1H NMRspectrum (CD3OD, 600 MHz) of 10. **Figure S24. **1H NMRspectrum (CDCl3, 600 MHz) of 11. **Figure S25. **13C NMRspectrum (CDCl3, 150 MHz) of 11. **Figure S26. **1H NMRspectrum (CDCl3, 600 MHz) of 12. **Figure S27. **13C NMRspectrum (CDCl3, 150 MHz) of 12. **Figure S28. **1H NMRspectrum (CDCl3, 600 MHz) of 13. **Figure S29. **13C NMRspectrum (CDCl3, 150 MHz) of 13. **Figure S30. **1H NMRspectrum (CDCl3, 600 MHz) of 14. **Figure S31. **13C NMRspectrum (CDCl3, 150 MHz) of 14.

## Data Availability

All data generated or analyzed during this study are included in this published article and its additional files.

## Abbreviations

^13^C NMR: Carbon-13 Nuclear Magnetic Resonance
^1^H NMR: Proton Nuclear Magnetic Resonance
CC_50_: 50% Cytotoxic concentration
*D. edulis*: *Dacroydes edulis*
DEM: Methanol extract of the stem bark of *D. edulis*
DEF: Hydroethanolic extract of the leaves of *D. edulis*
DMSO: Dimethyl sulfoxide
DCM: Dichloromethane
EDTA: Ethylenediaminetetraacetic acid
HNC: Herbier National du Cameroun
H_2_O: Water
IC_50_: Inhibitory concentration 50%
LD_50_: Lethal Dose 50
MeOH: Methanol
NMR: Nuclear Magnetic Resonance spectroscopy
RI: Resistance index
SD: Standard deviation
SI: Selectivity index
WHO: World Health Organization
