# Supplementary material for: In vitro antiplasmodial activity and toxicological profile of extracts, fractions and chemical constituents of leaves and stem bark from Dacryodes edulis (Burseraceae)
Source: BMC Complement Med Ther. 2023 Jun 27;23:211. doi: 10.1186/s12906-023-03957-2 (PMC10303846; doi:10.1186/s12906-023-03957-2)
Supplement: Supplementary file 1 — Additional file 1: Figure S1. HR-ESI mass spectrum of 1. Figure S2. 1H NMR spectrum(Pyridin-d5, 600 MHz) of 1. Figure S3. 13C NMR spectrum (Pyridin-d5, 150 MHz) of 1. Figure S4. ESI mass spectrum of2. Figure S5. 1H NMR spectrum (CDCl3, 600 MHz) of 2. Figure S6. 13C NMR spectrum (CDCl3, 150 MHz) of 2. Figure S7. 1H NMR spectrum(Pyridin-d5, 600 MHz) of 3. Figure S8. 1H NMR spectrum(Pyridin-d5, 600 MHz) of 4. Figure S9. 13C NMR spectrum(Pyridin-d5, 150 MHz) of 4. Figure S10. HR-ESI mass spectrum of 5. Figure S11. 1H NMRspectrum (CD3OD, 600 MHz) of 5. Figure S12. 13C NMRspectrum (CD3OD, 150 MHz) of 5. Figure S13. 1H NMRspectrum (CD3OD, 600 MHz) of 6. Figure S14. 13C NMRspectrum (CD3OD, 150 MHz) of 6. Figure S15. HR-ESI mass spectrum of 7. Figure S16. 1H NMRspectrum (CDCl3, 600 MHz) of 7. Figure S17. 13C NMRspectrum (CDCl3, 150 MHz) of 7. Figure S18. 1H NMRspectrum (CDCl3, 600 MHz) of 8. Figure S19. 13C NMRspectrum (CDCl3, 150 MHz) of 8. Figure S20. HR-ESI mass spectrum of 9. Figure S21. 1H NMRspectrum (CDCl3, 600 MHz) of 9. Figure S22. 13C NMRspectrum (CDCl3, 150 MHz) of 9. Figure S23. 1H NMRspectrum (CD3OD, 600 MHz) of 10. Figure S24. 1H NMRspectrum (CDCl3, 600 MHz) of 11. Figure S25. 13C NMRspectrum (CDCl3, 150 MHz) of 11. Figure S26. 1H NMRspectrum (CDCl3, 600 MHz) of 12. Figure S27. 13C NMRspectrum (CDCl3, 150 MHz) of 12. Figure S28. 1H NMRspectrum (CDCl3, 600 MHz) of 13. Figure S29. 13C NMRspectrum (CDCl3, 150 MHz) of 13. Figure S30. 1H NMRspectrum (CDCl3, 600 MHz) of 14. Figure S31. 13C NMRspectrum (CDCl3, 150 MHz) of 14. [file 12906_2023_3957_MOESM1_ESM.pdf]

ADDITIONAL FIILE

In vitro antiplasmodial activity and toxicological profile of extracts, fractions and chemical constituents of leaves and stem bark of *Dacryodes edulis* (Burseraceae)

By

Kevine Johane Jumeta Dongmo [1], Mariscal Brice Tchatat Tali [2], Yannick Stéphane Fotsing Fongang [3,*], Pierre Leonel K. Tafokeu Taguimjeu [1], Donald Ulrich Kenou Kagho [1], Gabin Thierry Bitchagno [4], Bruno Ndjakou Lenta [5,*], Fabrice Boyom Fekam [2], Norbert Sewald [6], Silvère Augustin Ngouela [1]

[1] Department of Organic Chemistry, Faculty of Science, University of Yaoundé I, P.O. Box 812 Yaoundé, Yaoundé, Cameroon (jumetakevine@yahoo.fr; taguimjeuleonel@outlook.fr; dkaghokenou@yahoo.fr)

[2] Antimicrobial and Biocontrol Agents Unit, Faculty of Science, University of Yaoundé I, P.O. Box 812 Yaoundé, Yaoundé, Cameroon (b.tchatat@yahoo.com; tchatatb7@gmail.com)

[3] Department of Chemistry, Higher Teachers' Training College, The University of Maroua, P.O. Box 55 Maroua, Maroua, Cameroon (fongangfys@yahoo.fr)

[4] Department of Chemistry, University of Dschang, P.O. Box 67 Dschang, Cameroon (bmgt198716@ymail.com)

[5] Department of Chemistry, Higher Teacher Training College, University of Yaoundé I, P.O. Box 47 Yaoundé, Yaoundé, Cameroon (lentabruno@yahoo.fr)

[6] Department of Chemistry, Bielefeld University, P.O. Box 100131, 33501, Bielefeld, Germany (Norbert.sewald@uni-bielefeld.de)

*** Corresponding Authors** : Yannick Stéphane Fotsing Fongang (fongangfys@yahoo.fr; Orcid number: 0000-0002-9311-9945) and Bruno Ndjakou Lenta (lentabruno@yahoo.fr)

Table of Contents

Figure S1: HR-ESI mass spectrum of **1**......3

Figure S2: ^{1}H NMR spectrum (Pyridin-d_5, 600 MHz) of **1**......3

Figure S3: ^{13}C NMR spectrum (Pyridin-d_5, 150 MHz) of **1**......4

Figure S4: ESI mass spectrum of **2**......4

Figure S5: ^{1}H NMR spectrum ($CDCl_3$, 600 MHz) of **2**......5

Figure S6: ^{13}C NMR spectrum ($CDCl_3$, 150 MHz) of **2**......5

Figure S7: ^{1}H NMR spectrum (Pyridin-d_5, 600 MHz) of **3**......6

Figure S8: ^{1}H NMR spectrum (Pyridin-d_5, 600 MHz) of **4**......6

Figure S9: ^{13}C NMR spectrum (Pyridin-d_5, 150 MHz) of **4**......7

Figure S10: HR-ESI mass spectrum of **5**......7

Figure S11: ^{1}H NMR spectrum (CD_3OD, 600 MHz) of **5**......8

Figure S12: ^{13}C NMR spectrum (CD_3OD, 150 MHz) of **5**......8

Figure S13: ^{1}H NMR spectrum (CD_3OD, 600 MHz) of **6**......9

Figure S14: ^{13}C NMR spectrum (CD_3OD, 150 MHz) of **6**......9

Figure S15: HR-ESI mass spectrum of **7**......10

Figure S16: ^{1}H NMR spectrum ($CDCl_3$, 600 MHz) of **7**......10

Figure S17: ^{13}C NMR spectrum ($CDCl_3$, 150 MHz) of **7**......11

Figure S18: ^{1}H NMR spectrum ($CDCl_3$, 600 MHz) of **8**......11

Figure S19: ^{13}C NMR spectrum ($CDCl_3$, 150 MHz) of **8**......12

Figure S20: HR-ESI mass spectrum of **9**......12

Figure S21: ^{1}H NMR spectrum ($CDCl_3$, 600 MHz) of **9**......13

Figure S22: ^{13}C NMR spectrum ($CDCl_3$, 150 MHz) of **9**......13

Figure S23: ^{1}H NMR spectrum (CD_3OD, 600 MHz) of **10**......14

Figure S24: ^{1}H NMR spectrum ($CDCl_3$, 600 MHz) of **11**......14

Figure S25: ^{13}C NMR spectrum ($CDCl_3$, 150 MHz) of **11**......15

Figure S26: ^{1}H NMR spectrum ($CDCl_3$, 600 MHz) of **12**......15

Figure S27: ^{13}C NMR spectrum ($CDCl_3$, 150 MHz) of **12**......16

Figure S28: ^{1}H NMR spectrum ($CDCl_3$, 600 MHz) of **13**......16

Figure S29: ^{13}C NMR spectrum ($CDCl_3$, 150 MHz) of **13**......17

Figure S30: ^{1}H NMR spectrum ($CDCl_3$, 600 MHz) of **14**......17

Figure S31: ^{13}C NMR spectrum ($CDCl_3$, 150 MHz) of **14**......18

Lichexanthone (1)

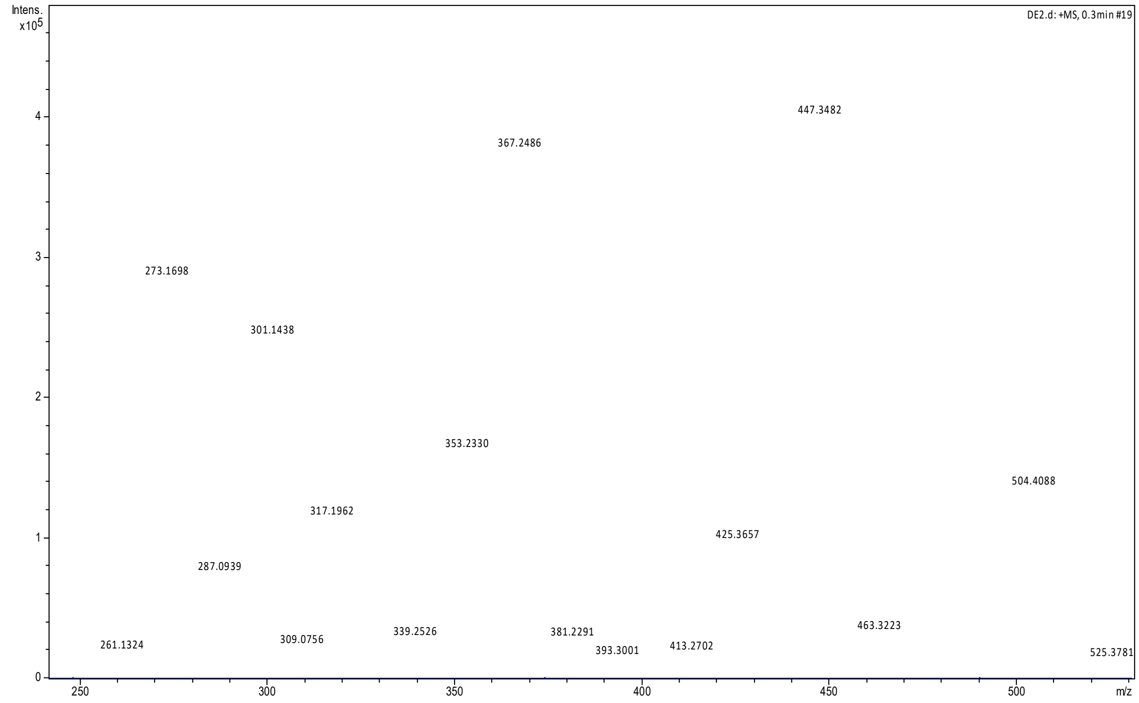

Figure S1: HR-ESI mass spectrum of **1**

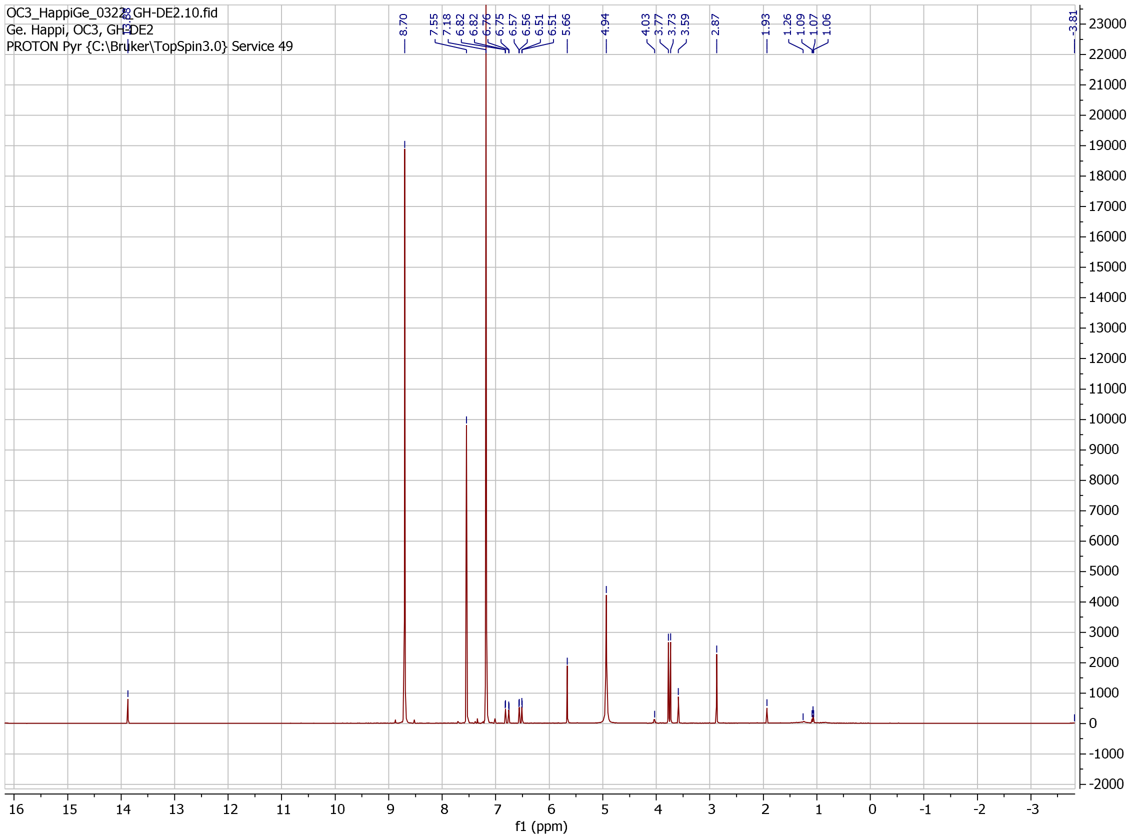

Figure S2: ^{1}H NMR spectrum (Pyridin-d_5, 600 MHz) of **1**

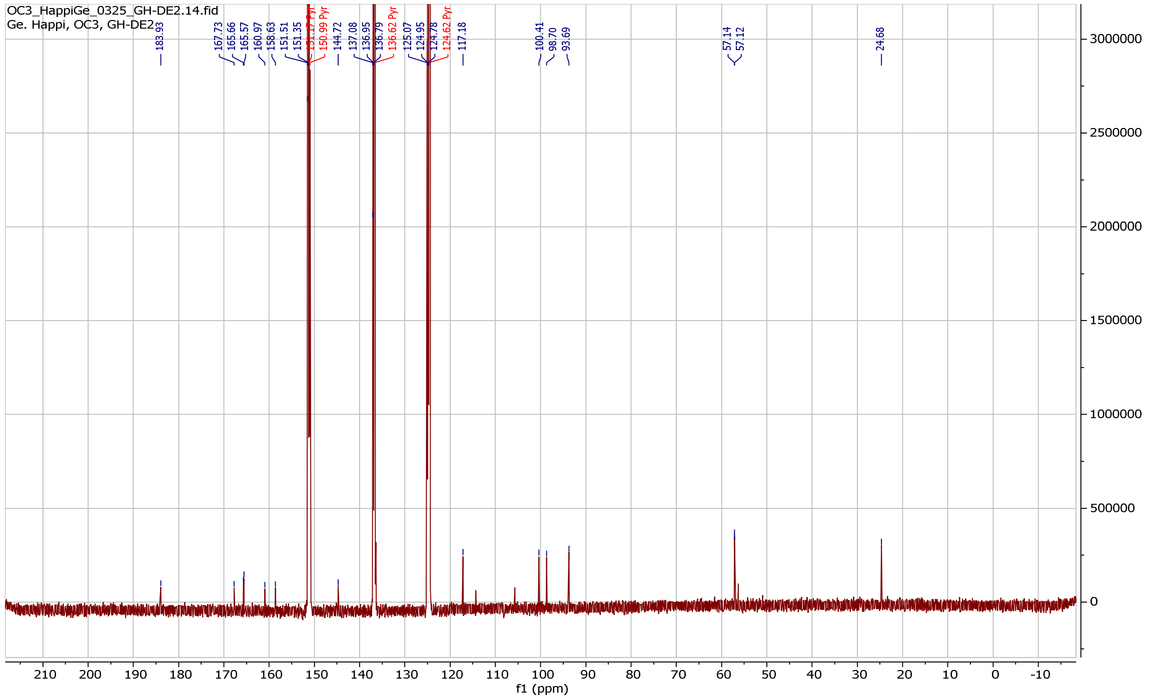

Figure S3: ^{13}C NMR spectrum (Pyridin-d_5, 150 MHz) of **1**

Griseoxanthone (2)

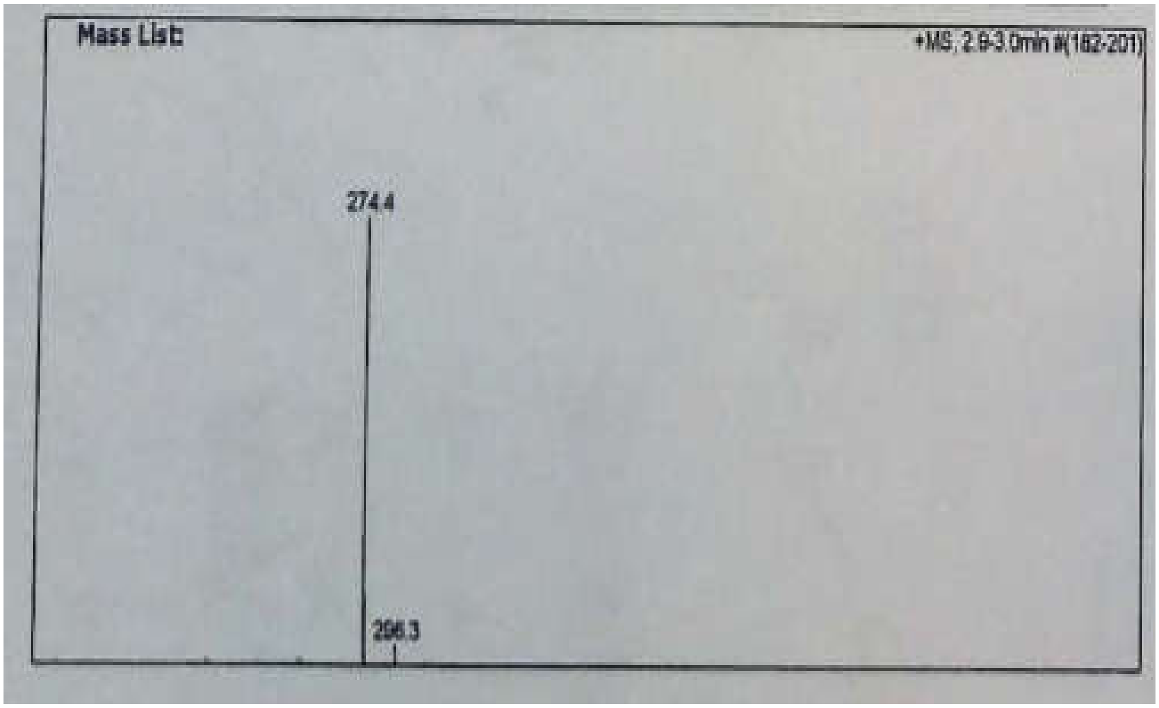

Figure S4: ESI mass spectrum of **2**

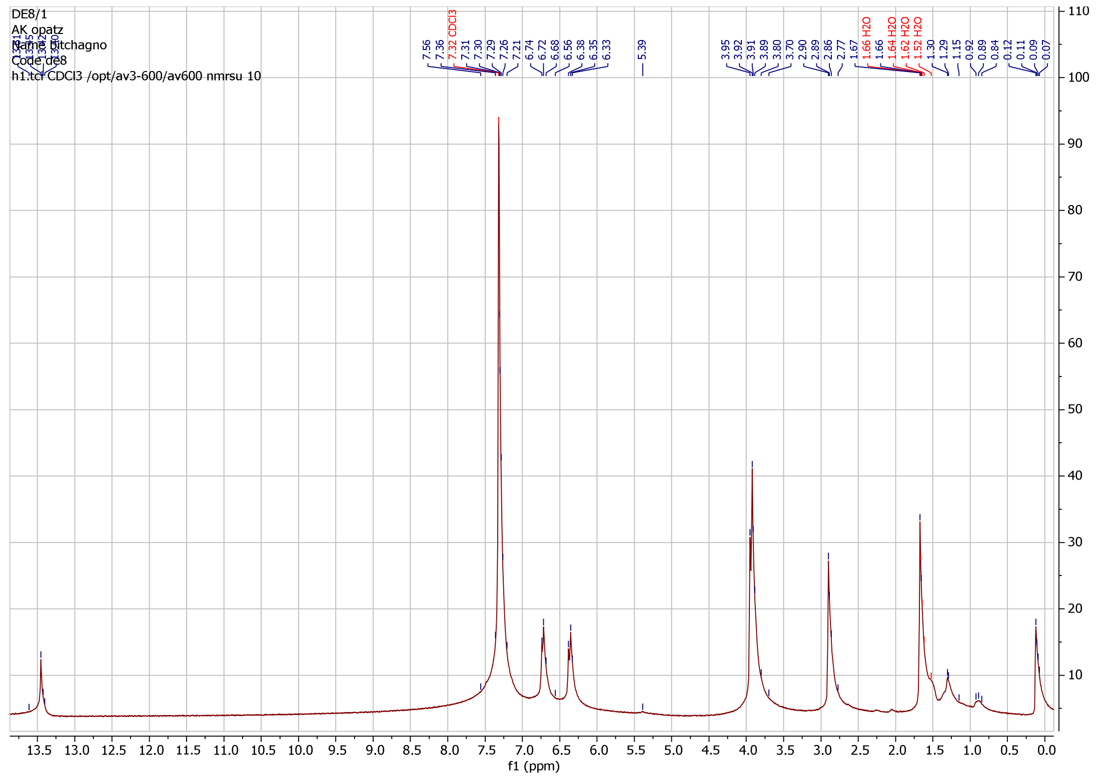

Figure S5: ^{1}H NMR spectrum ($CDCl_3$, 600 MHz) of **2**

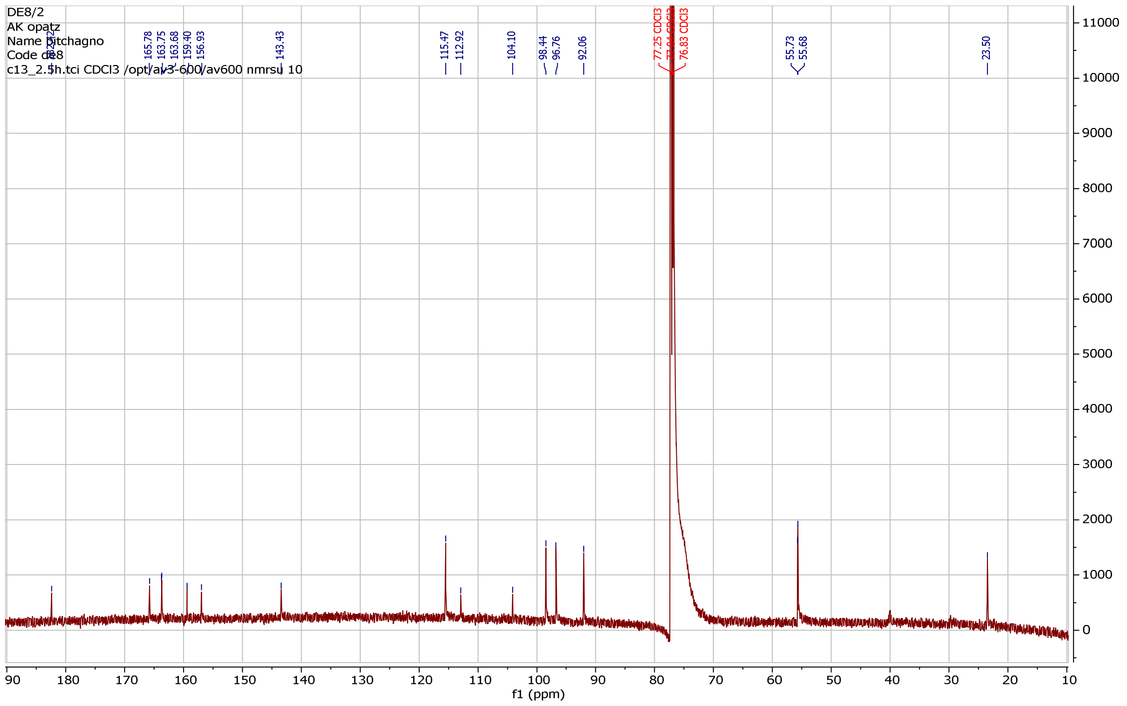

Figure S6: ^{13}C NMR spectrum ($CDCl_3$, 150 MHz) of **2**

3-3'-*O*-dimethylellargic acid (3)

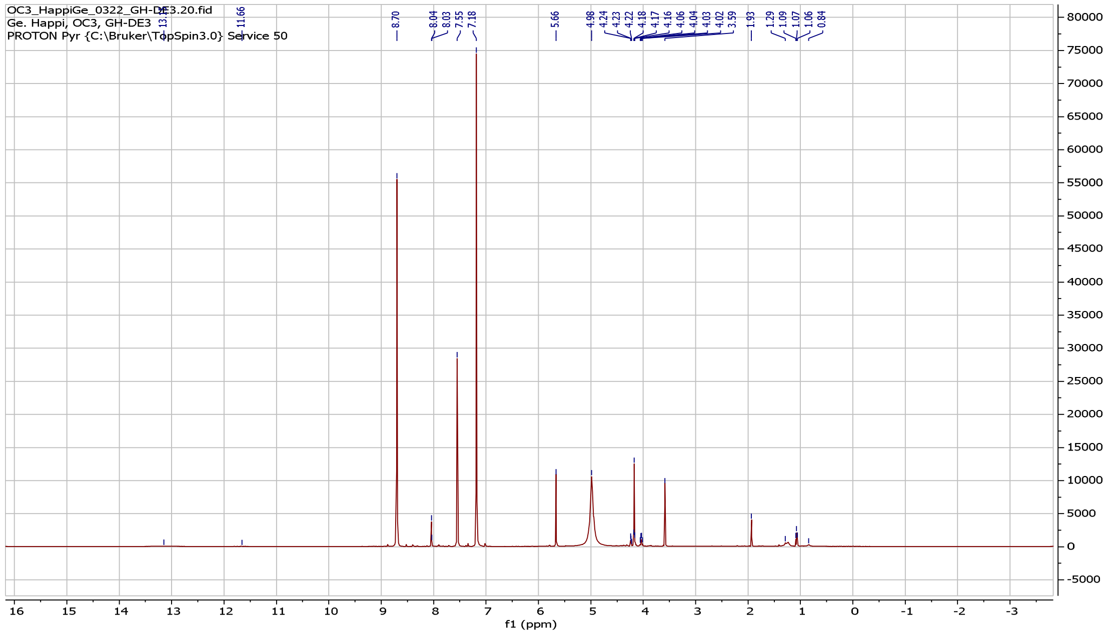

Figure S7: ^{1}H NMR spectrum (Pyridin-d_5, 600 MHz) of **3**

3,3′,4-tri-*O*-methylellargic (4)

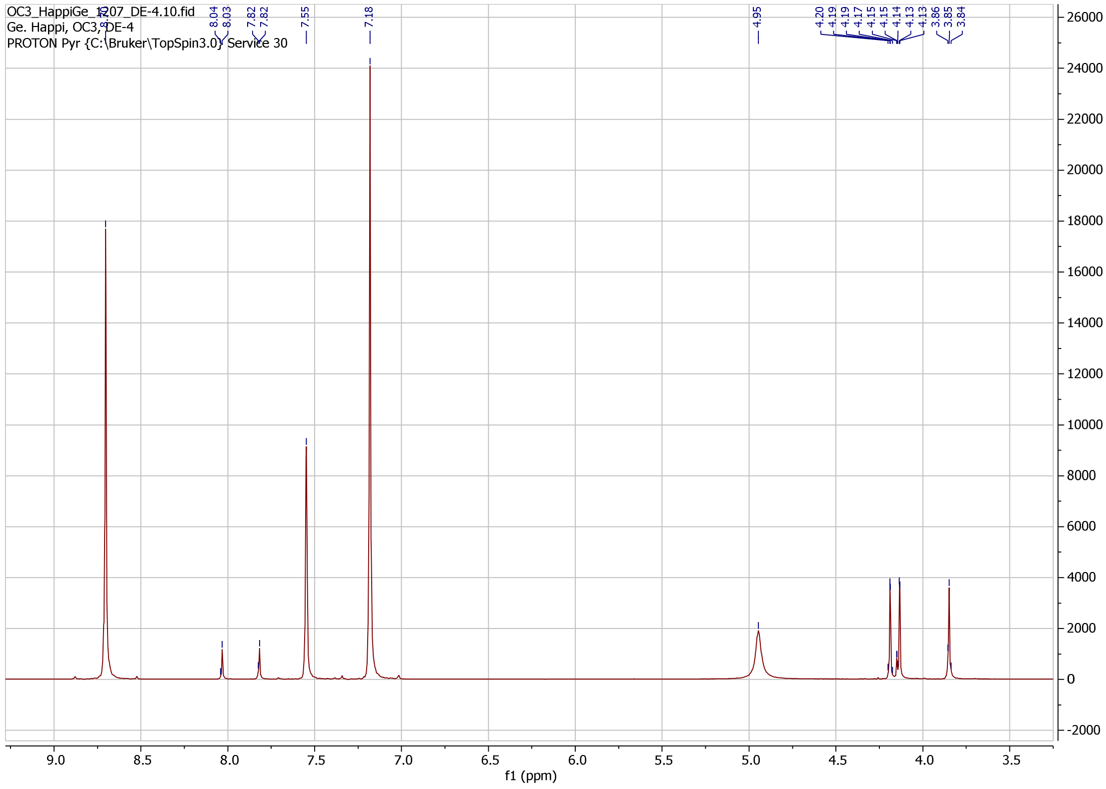

Figure S8: ^{1}H NMR spectrum (Pyridin-d_5, 600 MHz) of **4**

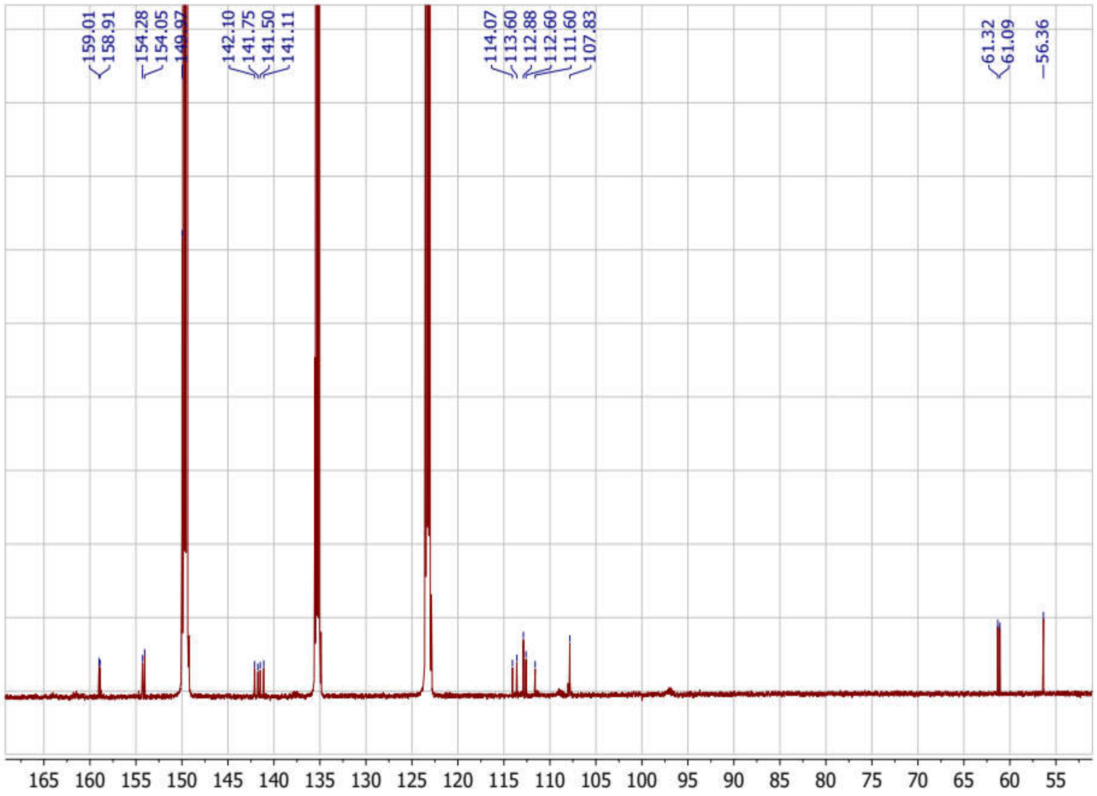

Figure S9: ^{13}C NMR spectrum (Pyridin-d_5, 150 MHz) of **4**

3,3″-di-*O*-methylellargic acid 4-*O*-(3″-galloyl)-*β*-*D*-xylopyranoside (5)

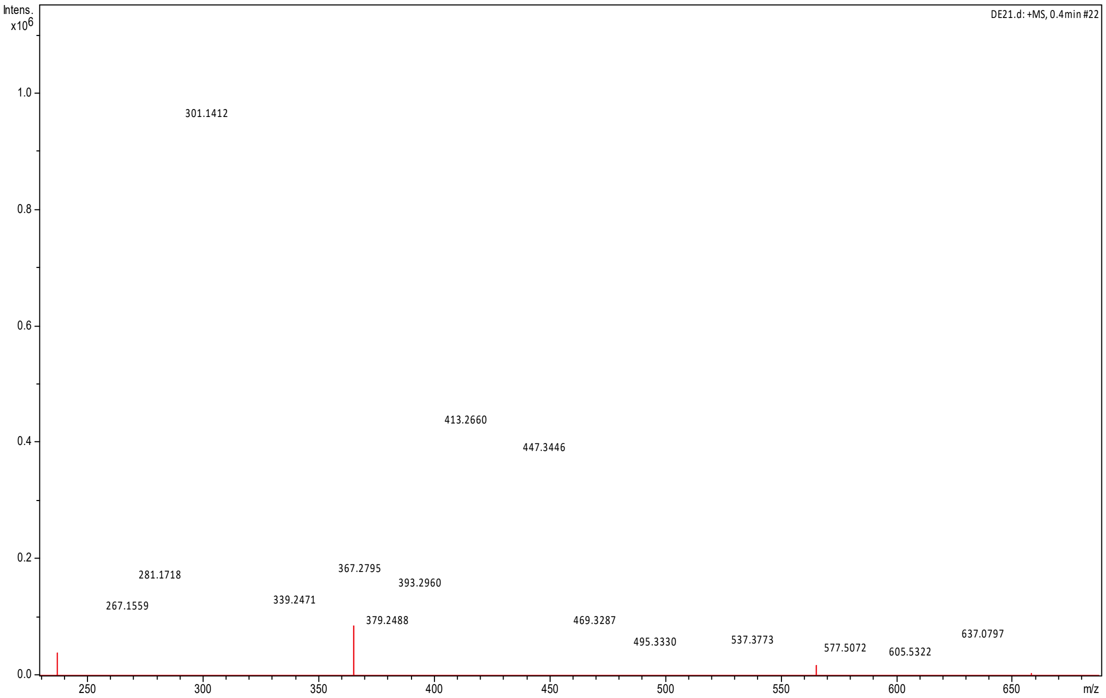

Figure S10: HR-ESI mass spectrum of **5**

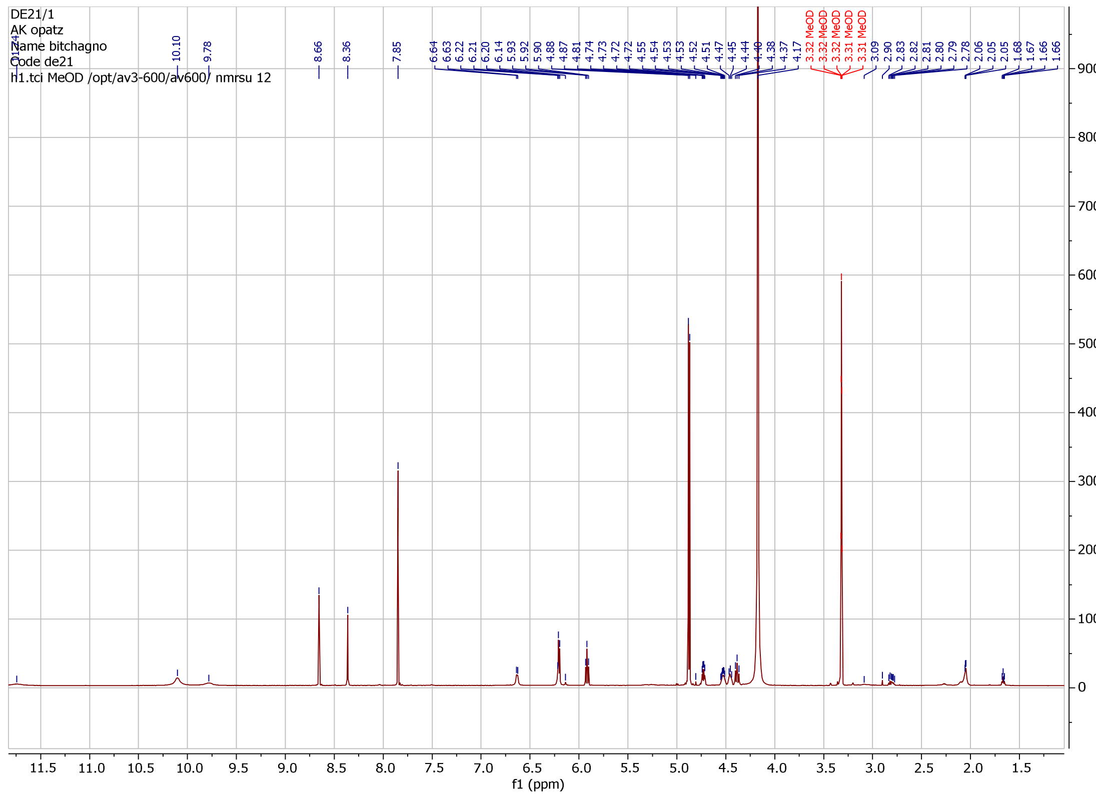

Figure S11: ^{1}H NMR spectrum (CD_3OD, 600 MHz) of **5**

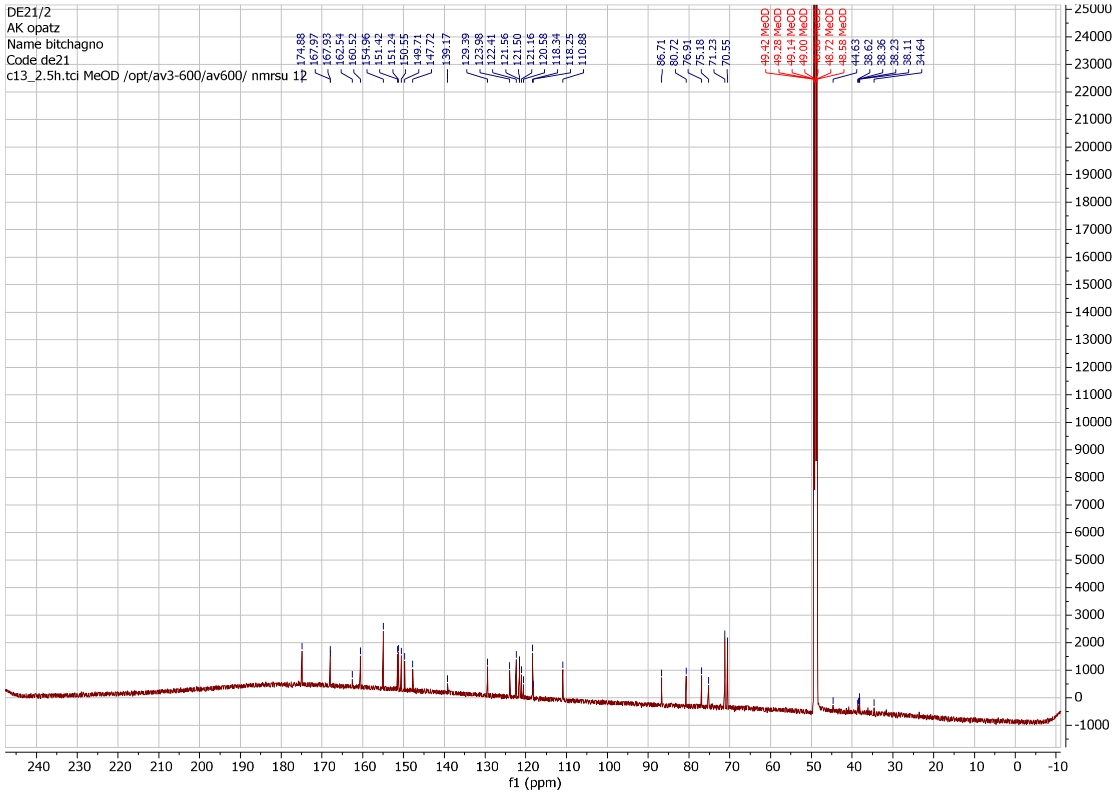

Figure S12: ^{13}C NMR spectrum (CD_3OD, 150 MHz) of **5**

3,4-dihydroxybenzoïc acid (6)

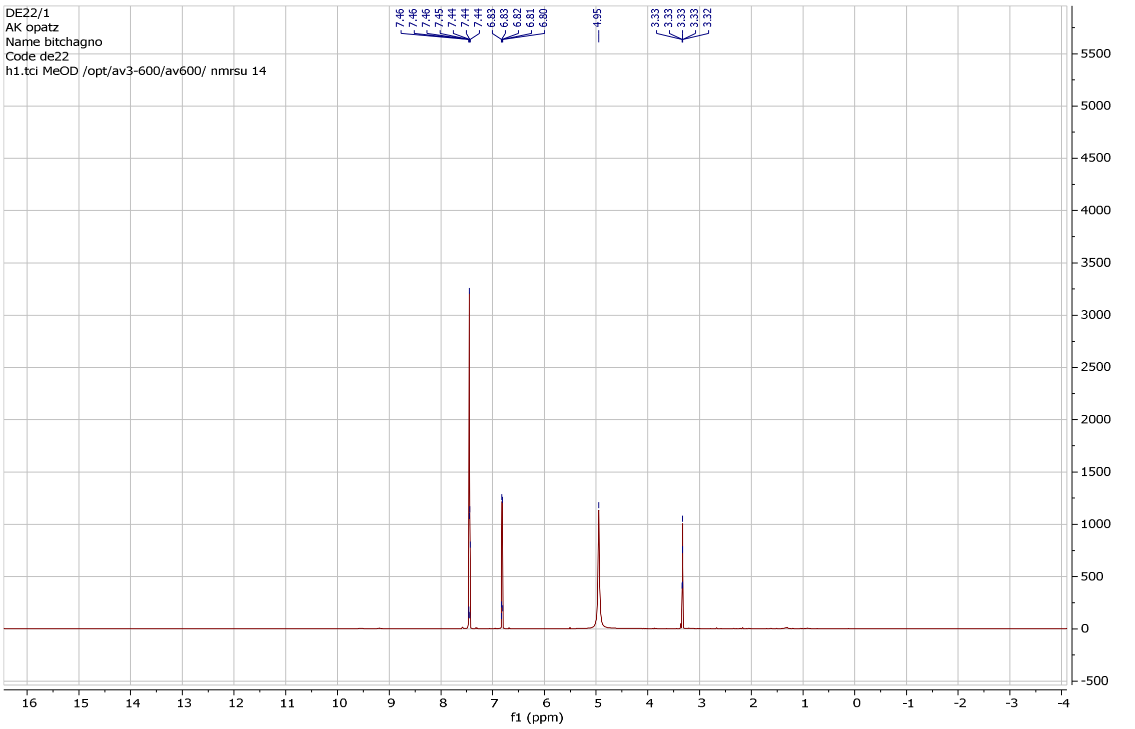

Figure S13: ^{1}H NMR spectrum (CD_3OD, 600 MHz) of **6**

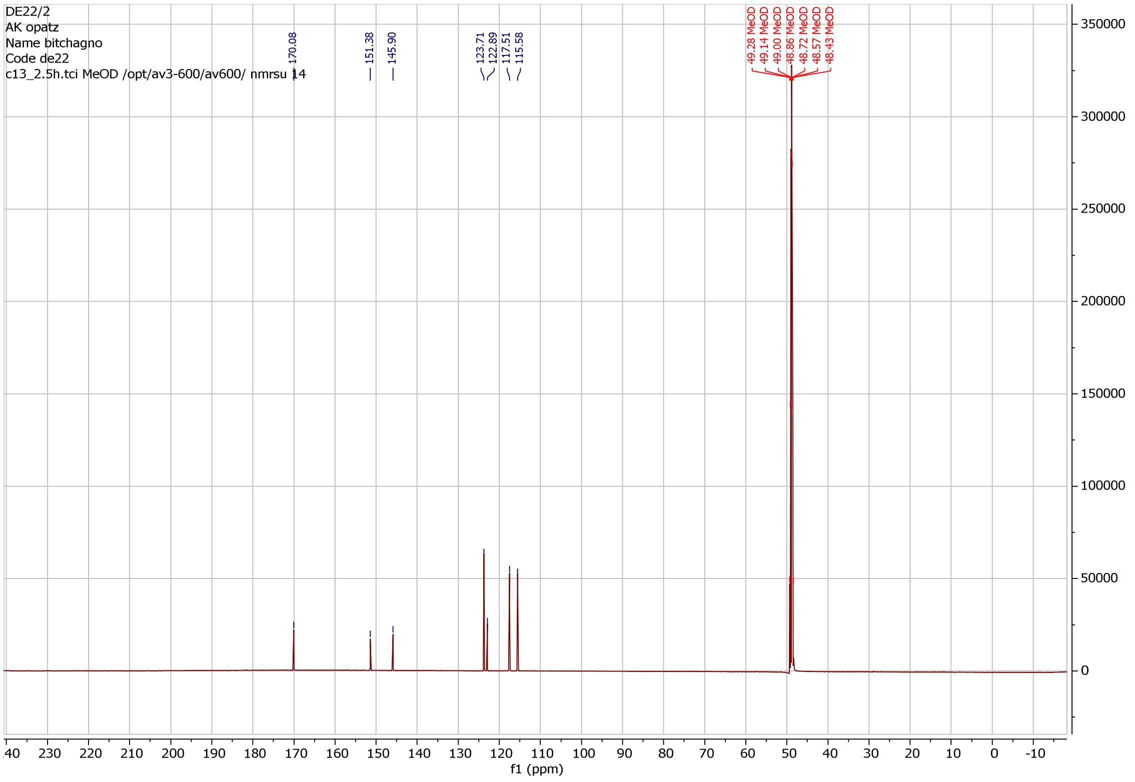

Figure S14: ^{13}C NMR spectrum (CD_3OD, 150 MHz) of **6**

Confluentic acid (7)

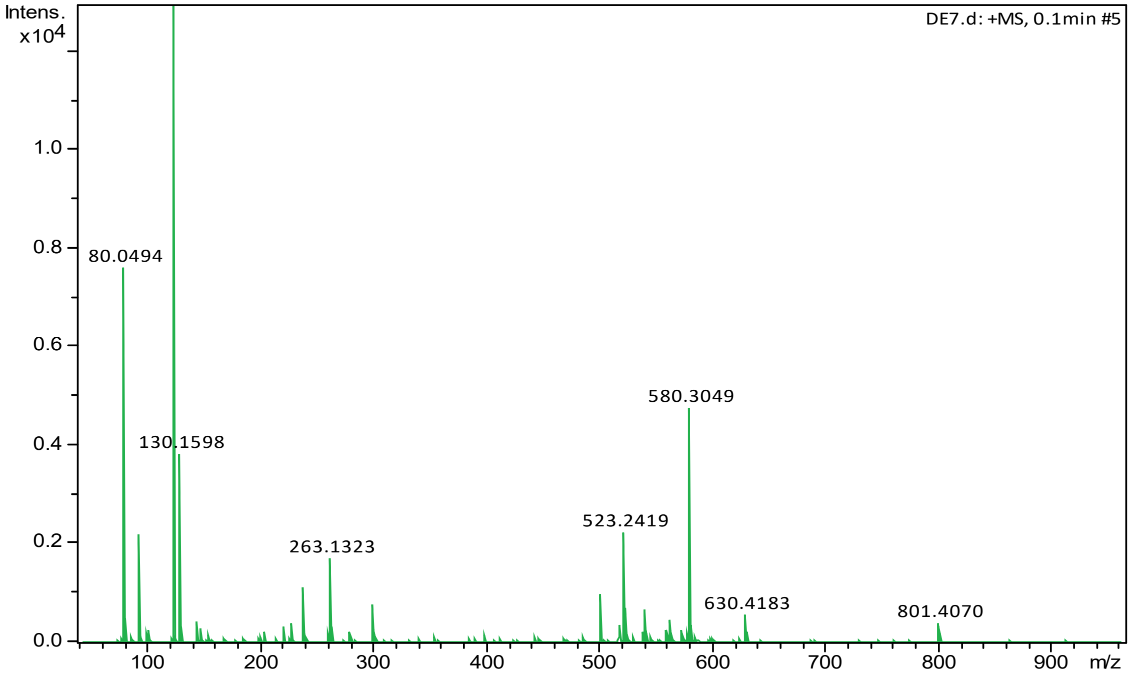

Figure S15: HR-ESI mass spectrum of **7**

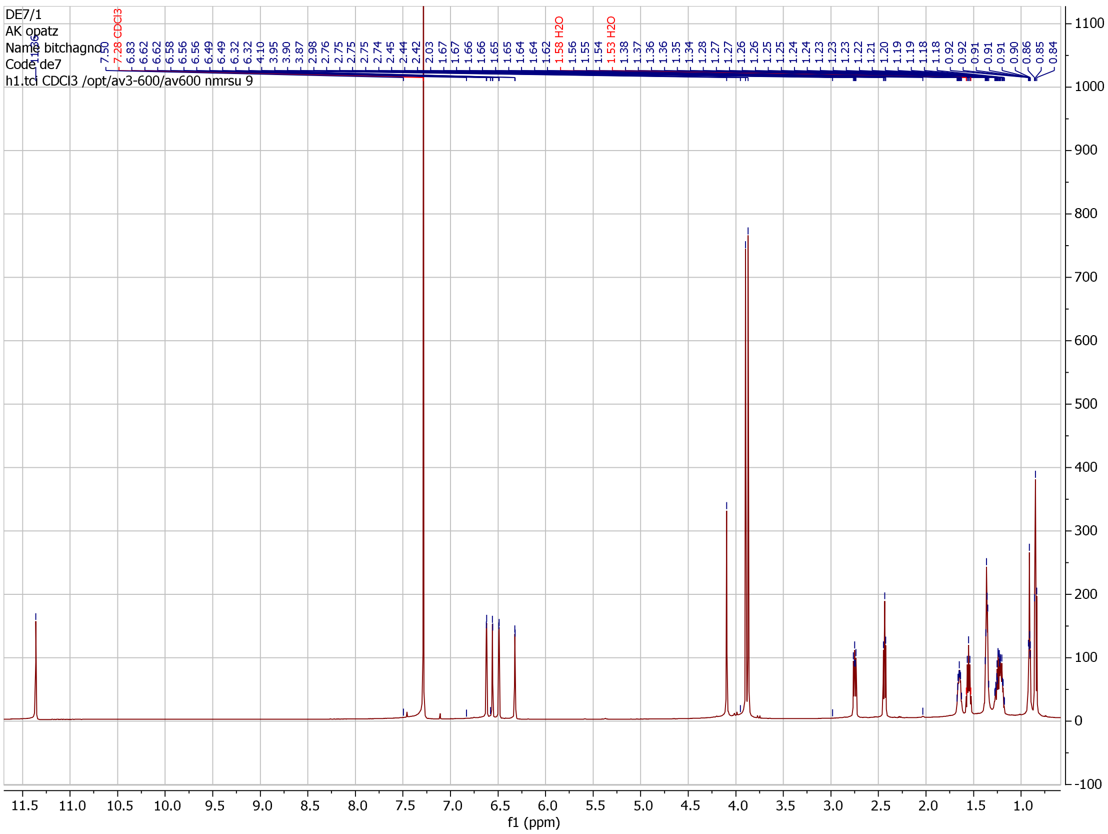

Figure S16: ^{1}H NMR spectrum ($CDCl_3$, 600 MHz) of **7**

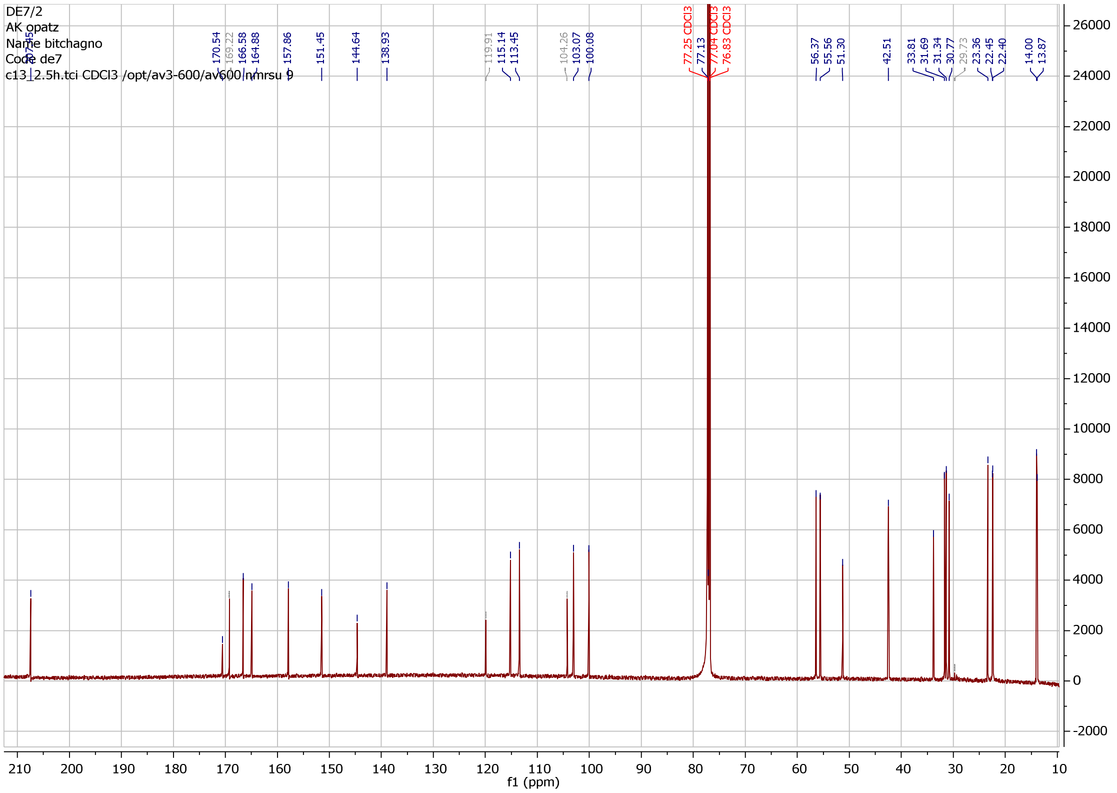

Figure S17: ^{13}C NMR spectrum ($CDCl_3$, 150 MHz) of **7**

Glyceryl-1-tetracosanoate (8)

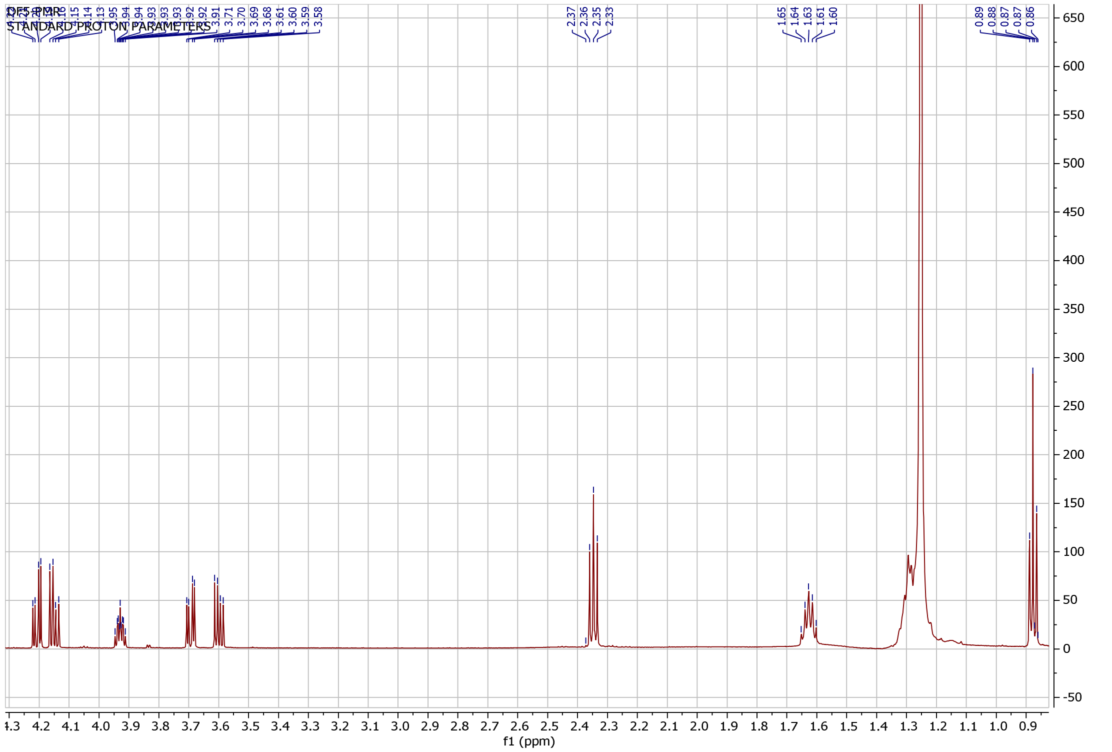

Figure S18: ^{1}H NMR spectrum ($CDCl_3$, 600 MHz) of **8**

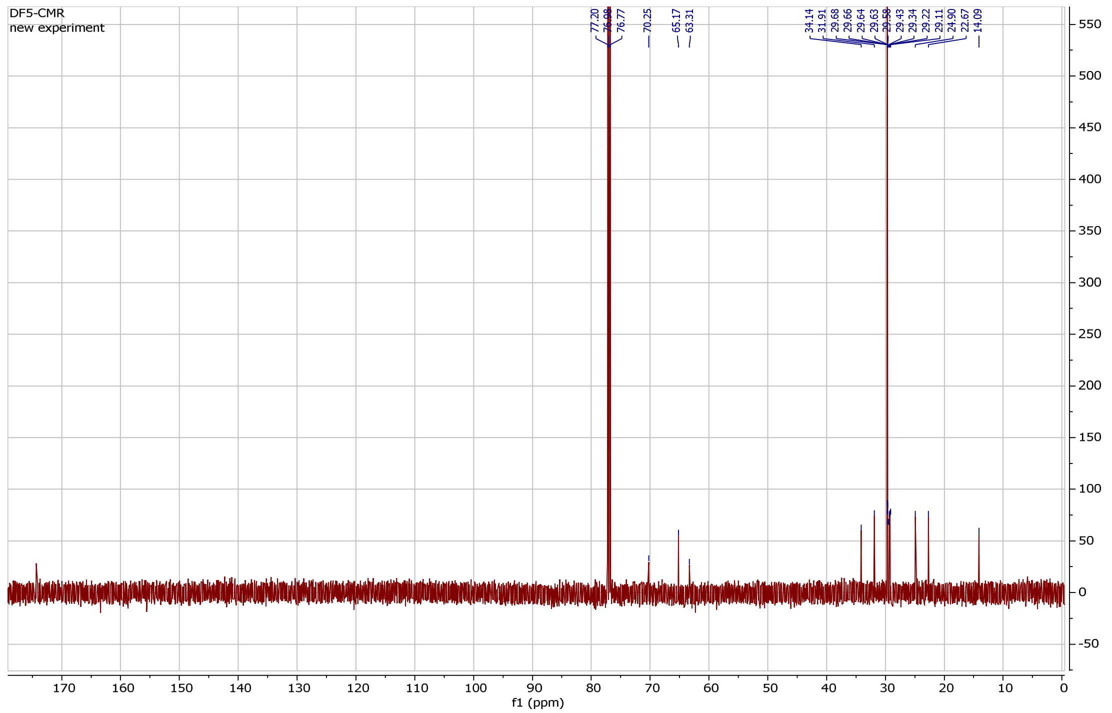

Figure S19: ^{13}C NMR spectrum ($CDCl_3$, 150 MHz) of **8**

Auranthiamide acetate (9)

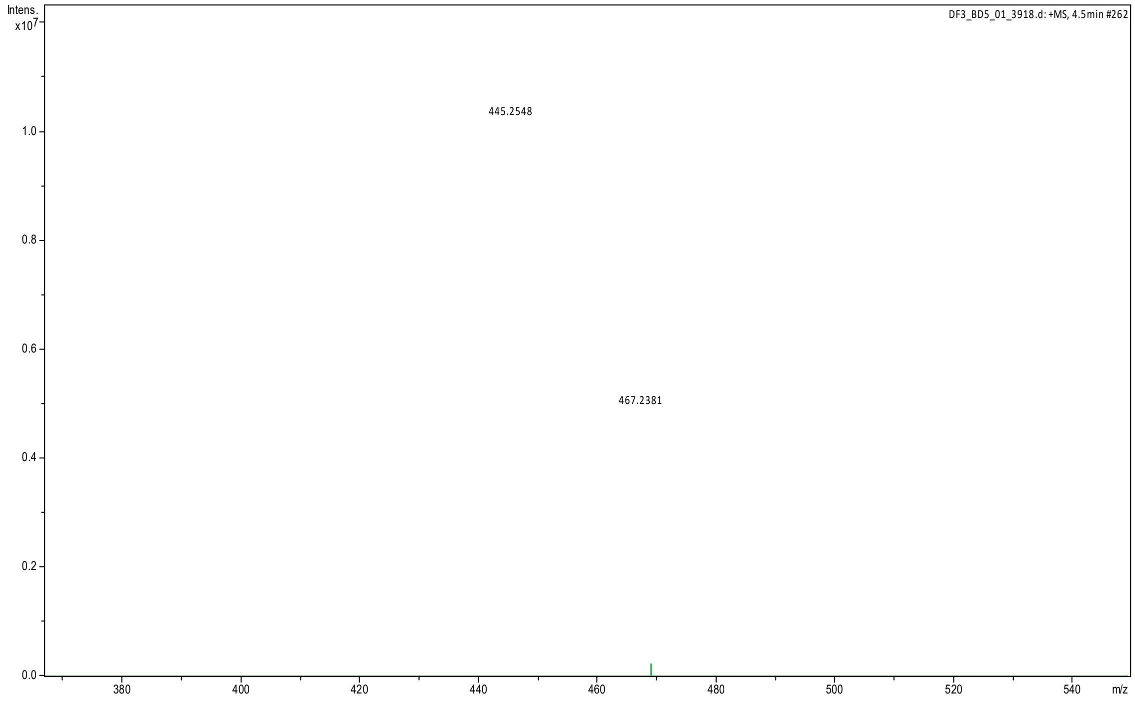

Figure S20: HR-ESI mass spectrum of **9**

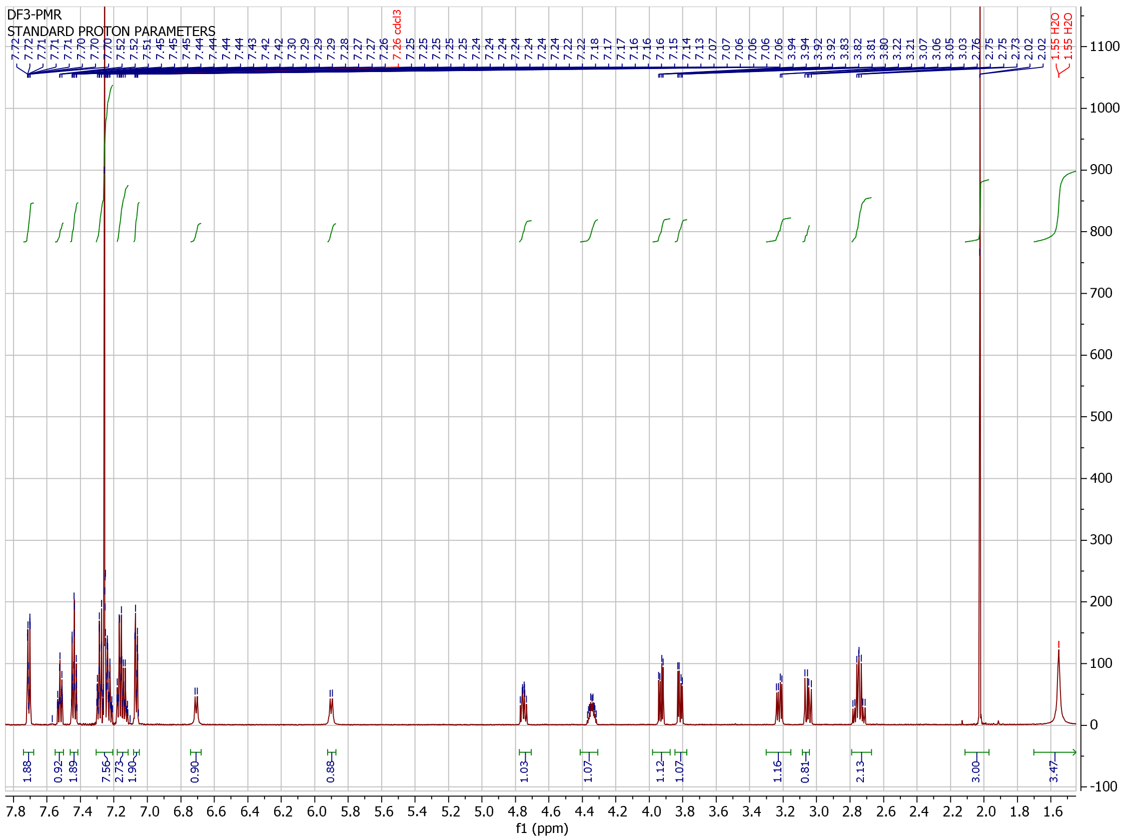

Figure S21: ^{1}H NMR spectrum ($CDCl_3$, 600 MHz) of **9**

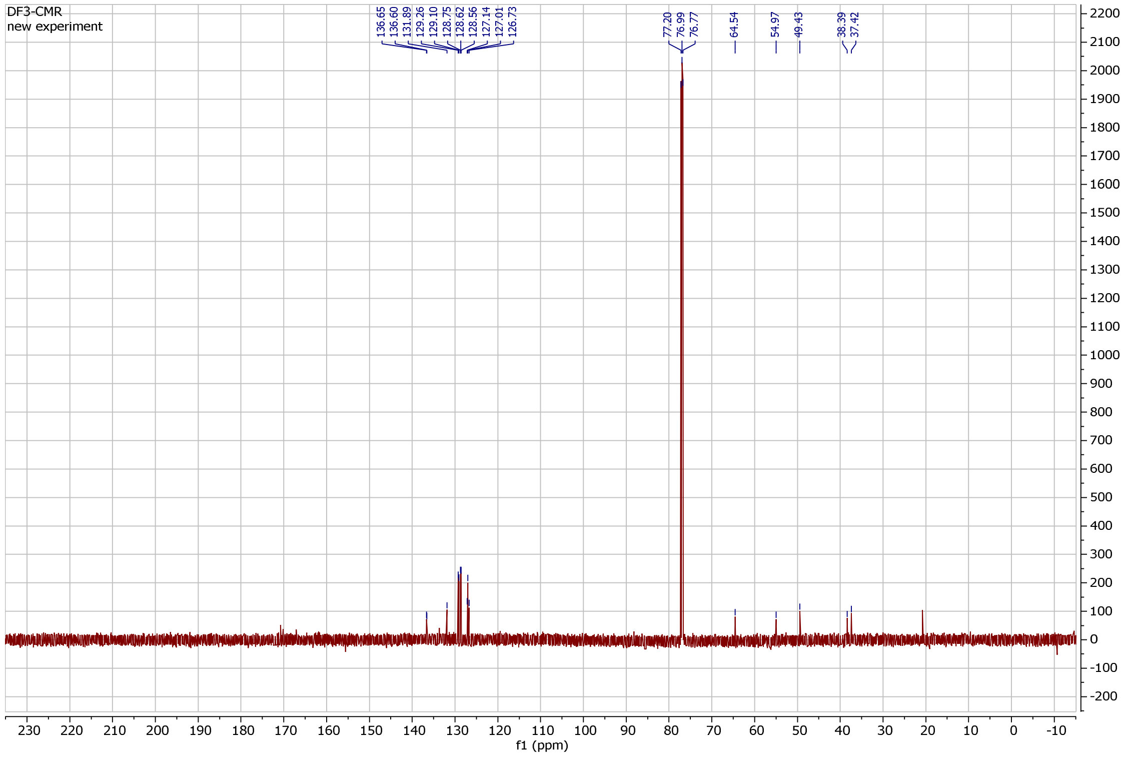

Figure S22: ^{13}C NMR spectrum ($CDCl_3$, 150 MHz) of **9**

Ethyl gallate (10)

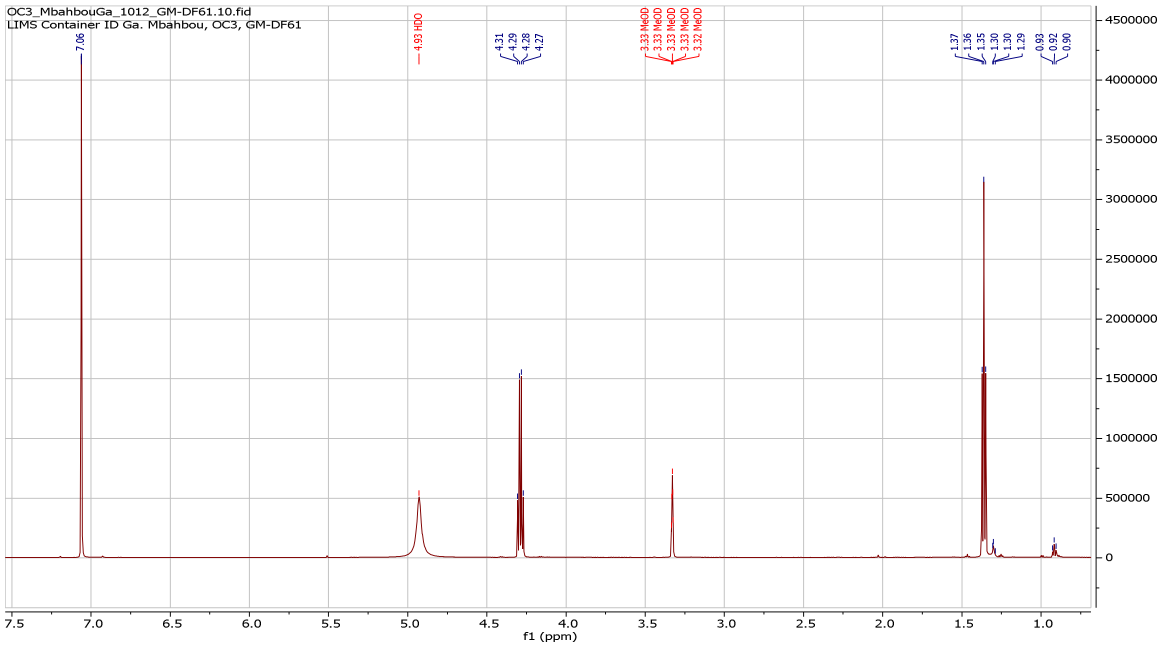

Figure S23: ^{1}H NMR spectrum (CD_3OD, 600 MHz) of **10**

β- amyrinacetate (11)

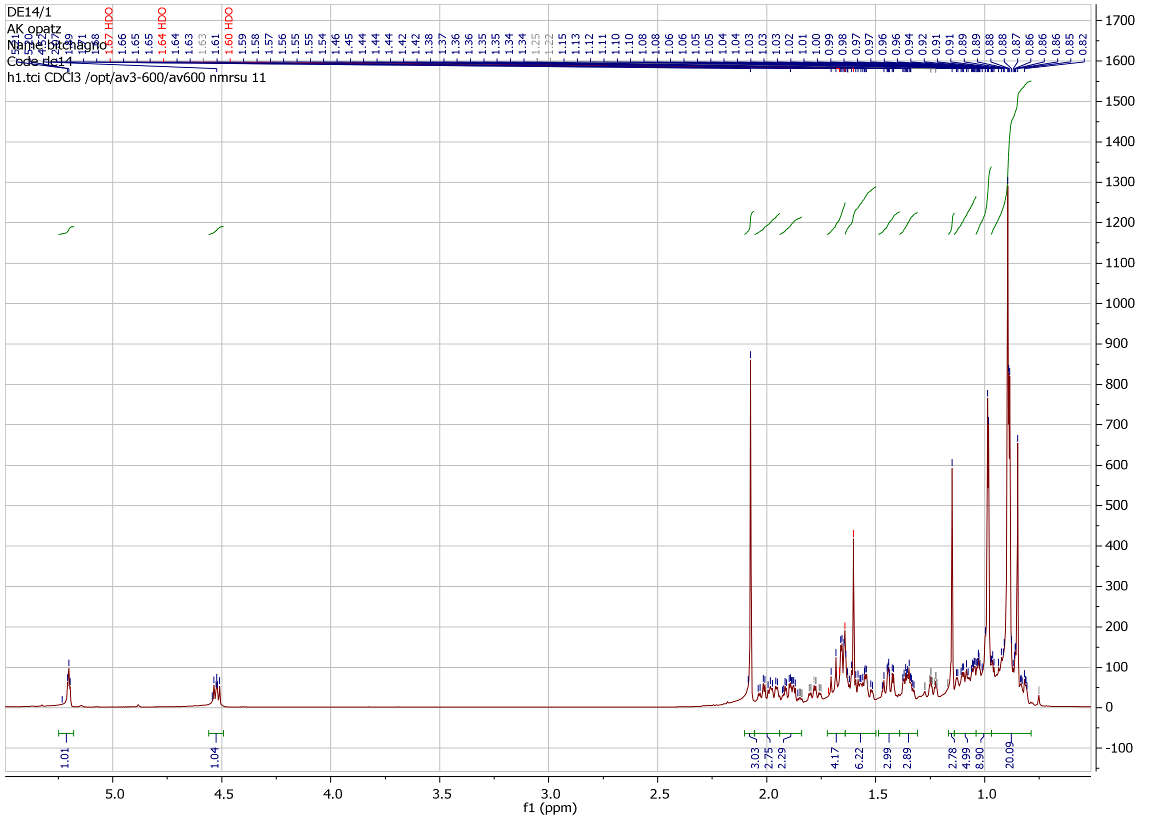

Figure S24: ^{1}H NMR spectrum ($CDCl_3$, 600 MHz) of **11**

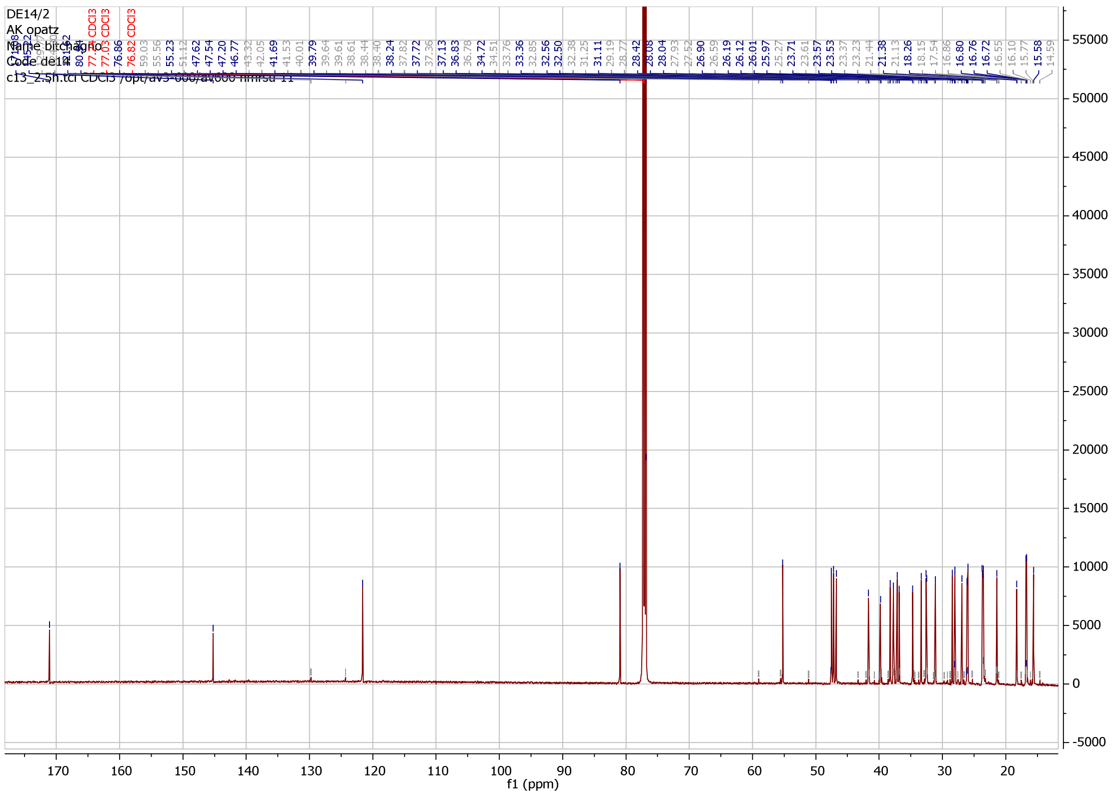

Figure S25: ^{13}C NMR spectrum ($CDCl_3$, 150 MHz) of **11**

***β*- amyrin (12)**

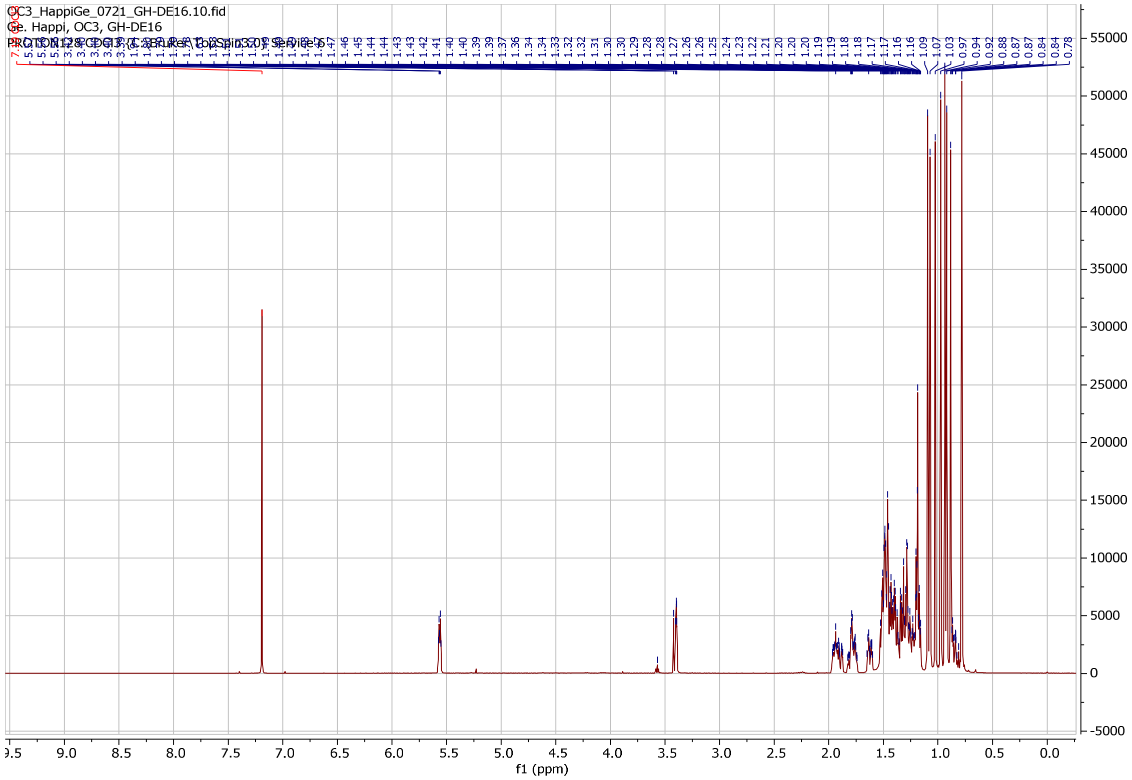

Figure S26: ^{1}H NMR spectrum ($CDCl_3$, 600 MHz) of **12**

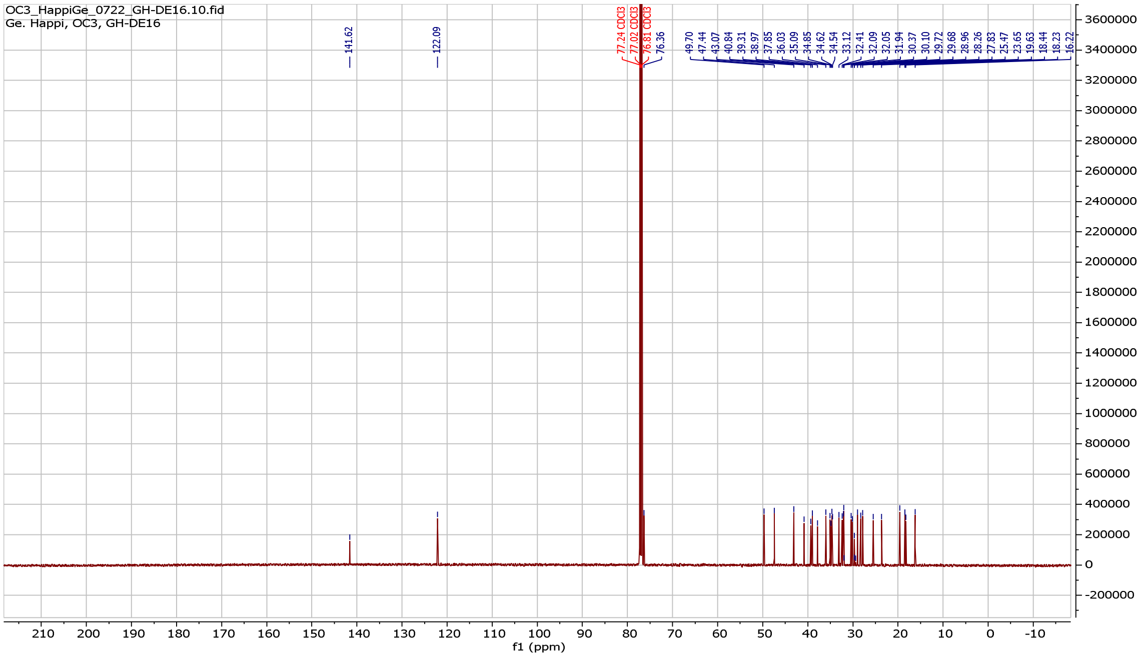

Figure S27: ^{13}C NMR spectrum ($CDCl_3$, 150 MHz) of **12**

3-Oxo-7,24*Z*-tirucalladien-26-oic acid (13)

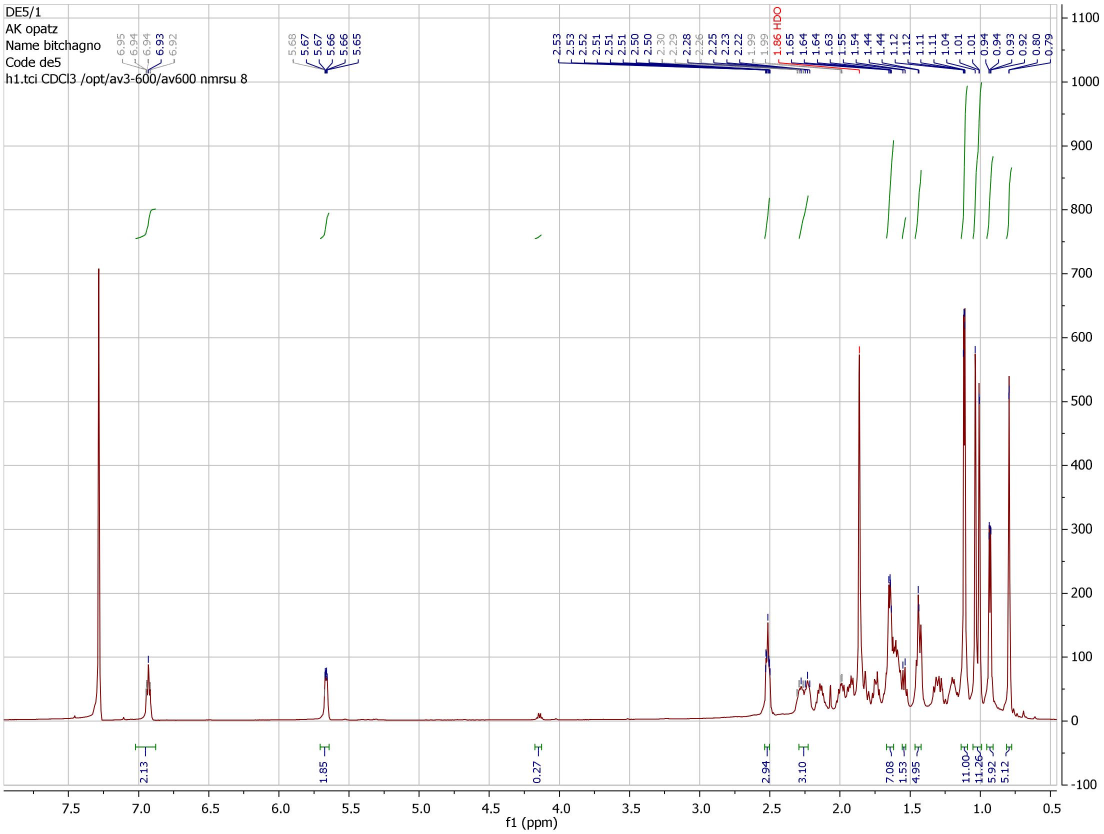

Figure S28: ^{1}H NMR spectrum ($CDCl_3$, 600 MHz) of 13

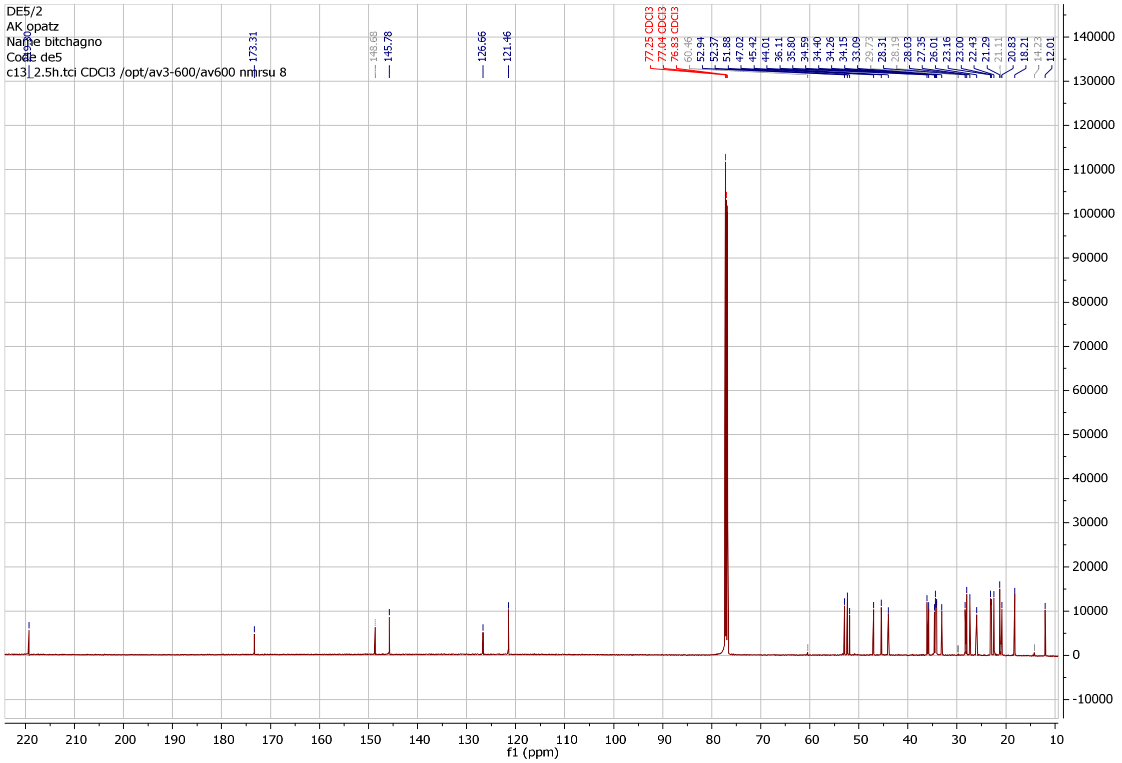

Figure S29: ^{13}C NMR spectrum ($CDCl_3$, 150 MHz) of **13**

Mixture of *β*-and *α*- amyrin (14)

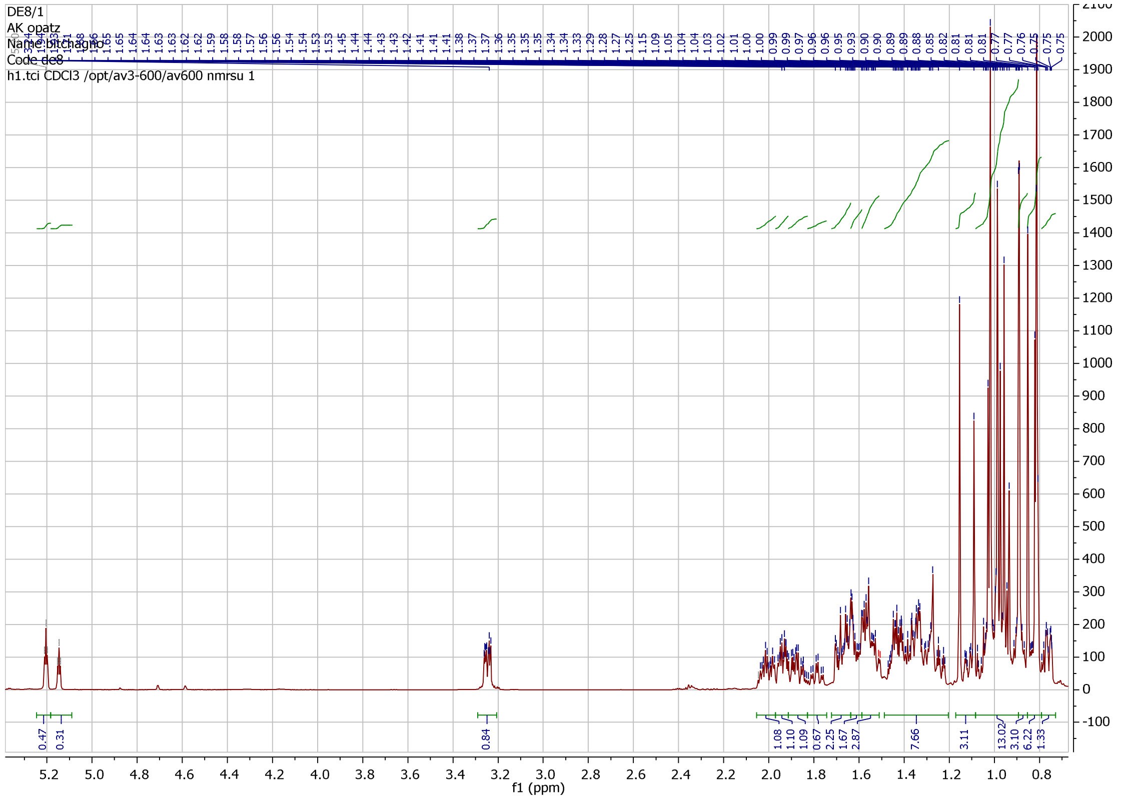

Figure S30: ^{1}H NMR spectrum ($CDCl_3$, 600 MHz) of **14**

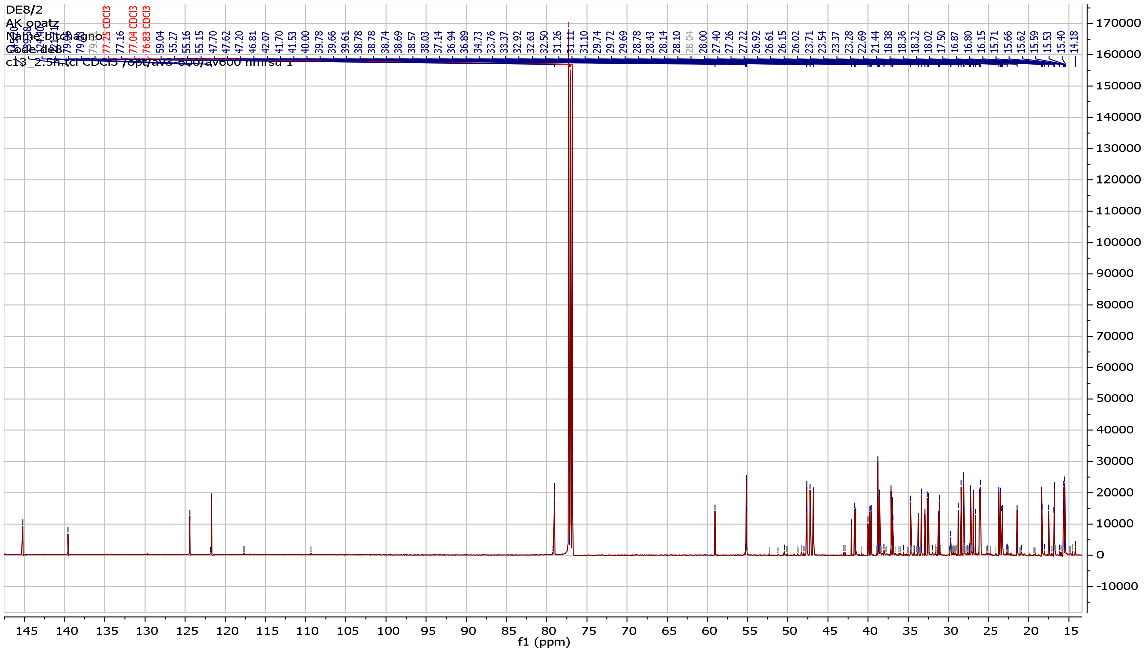

Figure S31: ^{13}C NMR spectrum ($CDCl_3$, 150 MHz) of **14**
